# Subclass III SnRK2 Kinases Coordinate Starch and Storage Protein Synthesis During Maize Kernel Development

**DOI:** 10.1111/pbi.70487

**Published:** 2025-12-08

**Authors:** Yayun Wang, Tiandan Long, Changqing Mao, Shan Feng, Qiang Liao, Yufeng Hu, Junjie Zhang, Hanmei Liu, Yinghong Liu, Xiujun Fan, Lei Gao, Canmei Cun, Binjie Xu, Daqiu Zhao, Jing Wang, Yubi Huang, Yangping Li

**Affiliations:** ^1^ State Key Laboratory of Crop Gene Exploration and Utilization in Southwest China Sichuan Agricultural University Chengdu Sichuan China; ^2^ Crop Research Institute Hunan Academy of Agricultural Sciences Changsha Hunan China; ^3^ Industrial Crop Research Institute Sichuan Academy of Agricultural Sciences Chengdu Sichuan China; ^4^ College of Life Science Sichuan Agricultural University Chengdu Sichuan China; ^5^ Maize Research Institute Sichuan Agricultural University Chengdu Sichuan China; ^6^ Plant Protection and Inspection Station, Yulong Bureau of Agriculture and Rural Affairs Lijiang Yunnan China; ^7^ Innovative Institute of Chinese Medicine and Pharmacy Chengdu University of Traditional Chinese Medicine Chengdu Sichuan China; ^8^ College of Horticulture and Landscape Architecture Yangzhou University Yangzhou Jiangsu China; ^9^ College of Agronomy Sichuan Agricultural University Chengdu Sichuan China

**Keywords:** kernel, maize, starch, storage proteins, subclass III SnRK2s

## Abstract

Enhancing both starch and protein accumulation is a key strategy for improving maize yield and quality. Achieving this goal requires an in‐depth understanding of the regulatory mechanisms that integrate these pathways. Here, we demonstrate that functionally redundant subclass III SNF1‐related protein kinase 2s (SnRK2s) act as central regulators that orchestrate starch synthesis and storage protein accumulation in maize kernels, with ZmSnRK2.10 playing a predominant role. Higher‐order SnRK2s mutants lacking ZmSnRK2.10 exhibit defective kernel development, with drastically reduced starch and storage protein content. Mechanistically, ZmSnRK2.10 directly phosphorylates starch synthesis‐related enzymes, such as Bt1, enhancing their activities and thereby boosting endosperm starch synthesis. Moreover, it indirectly promotes storage protein synthesis in both endosperm and embryo through modulating the phosphorylation status of downstream transcription factors, specifically Opaque‐2 and ZmbZIP75, respectively. Interestingly, our study reveals that ZmSnRK2.10 undergoes sequential activation: initially by sucrose in the endosperm during early kernel filling and subsequently by abscisic acid (ABA) in the embryo during later developmental stages. This spatiotemporal regulation suggests a mechanism facilitating coordinated control of these temporally linked processes. Notably, overexpression of *ZmSnRK2.10* leads to significant increases in both starch and protein content, as well as a higher vitreous endosperm ratio, thereby simultaneously enhancing maize yield and quality. Our study thus uncovers a previously unknown regulatory mechanism involving subclass III SnRK2s that govern storage functions in maize kernels and provides potential genetic resources for yield and quality improvement.

## 1 Introduction

Maize is a globally leading multipurpose cereal crop with rising demand for food, feed, and industrial applications (Erenstein et al. 2022). The maize kernel is the primary site for accumulating storage reserves, mainly starch and storage proteins (zeins and globulins), comprising 70% and 10% of grain dry weight, respectively. Starch and zeins are synthesized synchronously in the endosperm during endosperm filling (Keeling and Myers 2010; Yang et al. 2023). The interaction between these components affects endosperm texture, an important agronomic trait associated with kernel test weight, grain integrity at harvest, and resistance to insects and pathogens (Caballero‐Rothar 2019; Kljak et al. 2018). In contrast, globulins (GLBs) are produced in the embryo during late kernel development (Rivin and Grudt 1991), contributing 10%–20% of the total protein and serving as a key determinant of kernel lysine content (A. L. Kriz 1989, 1999; Zheng et al. 2019). Thus, the synthesis of starch and storage proteins largely determines both the yield and quality of maize.

Starch biosynthesis involves at least four key enzyme activities (Huang et al. 2021). ADP‐glucose pyrophosphorylase (AGPase) catalyzes the conversion of glucose‐1‐phosphate into ADP‐glucose (ADPG) in the cytosol, which is then transported to the endospermic amyloplast for glucan chain elongation (James et al. 2003). This transport is controlled by Brittle 1 (Bt1), a plastid membrane‐localized ADPG translocator (Kirchberger et al. 2007). In amyloplasts, starch synthesis occurs through the synergistic actions of starch synthases (SSs), starch branching enzymes (SBEs), and starch debranching enzymes (DBEs) (Hannah and Boehlein 2017). Zeins are categorized into four types with different molecular sizes: α (19 and 22 kD), β (15 kD), γ (16, 27, and 50 kD), and δ (10 and 18 kD) (Coleman and Larkins 1999; Esen 1987). α‐type zeins are encoded by multiple genes, whereas β‐, γ‐, and δ‐types are encoded by a single gene (Xu and Messing 2009). A classic endosperm‐specific transcription factor (TF), Opaque 2 (O2), positively regulates nearly all zein genes (Li et al. 2015; Schmidt et al. 1987; Zhan et al. 2018). GLBs consist of two members, globulin‐1 and globulin‐2, encoded by embryo‐specific *Glb1* and *Glb2* genes, respectively (A. L. Kriz 1989). The transcription of *Glb1* and *Glb2* is primarily activated by the combined action of abscisic acid (ABA) and Viviparous‐1 (VP1), a master TF involved in late embryo development and seed dormancy (Rivin and Grudt 1991; Zheng et al. 2019).

Phosphorylation plays an important role in regulating grain filling and kernel development (Walley et al. 2013). Over the past two decades, extensive phosphorylation of starch synthesis‐related enzymes (SSREs) during grain filling has been documented across various cereals (Chen et al. 2016; Ferrero et al. 2020; Grimaud et al. 2008; Mehrpouyan et al. 2021; Pang et al. 2018; Tetlow et al. 2004). This was initially observed in wheat endosperm, where all SBEs are phosphorylated in amyloplasts, leading to enhanced catalytic activity (Tetlow et al. 2004). Subsequent studies have confirmed a positive correlation between SSRE phosphorylation and enzyme activity in rice and maize (Mehrpouyan et al. 2021; Yu et al. 2023; Zhang, Zhu, et al. 2019). In maize endosperm, multiple SSREs, including Sh2 and Bt2 (AGPase large and small subunits), Bt1, GBSSI (Wx, granule‐bound SS), SSIIa, and all SBEs, undergo phosphorylation (Grimaud et al. 2008; Walley et al. 2013). However, the specific kinases responsible for this posttranslational modification and their functional significance remain largely unknown. Although zein genes are primarily regulated at the transcriptional level (Yang et al. 2023), O2 activity is modulated by dynamic phosphorylation changes throughout endosperm filling, with hyperphosphorylated O2 exhibiting reduced DNA binding capacity (Ciceri et al. 1997). A recent study suggests that under low sucrose conditions, SNF1‐related kinase1 (SnRK1) directly phosphorylates O2, negatively regulating its transactivation of downstream zein genes (Yang et al. 2024), indicating that O2 phosphorylation is tightly regulated by cellular sugar levels in maize endosperm.

SnRK2, a plant‐specific kinase family, is classified into three subclasses (I, II, and III). Subclass III SnRK2s are core components of ABA signaling, regulating ABA‐triggered processes such as seed dormancy, germination, stomatal closure, and various abiotic stress responses (Mao et al. 2020). Unexpectedly, several studies have also uncovered emerging roles of subclass III SnRK2s in plant growth and development under nonstress conditions (Belda‐Palazón et al. 2020; Fujii and Zhu 2009; Nakashima et al. 2009; Yoshida et al. 2019). For example, mutations in all three subclass III SnRK2s (*SnRK2.2/2.3/2.6*) in Arabidopsis cause defective seed development and altered leaf growth and metabolic balance under optimal conditions (Nakashima et al. 2009; Yoshida et al. 2019). There are four subclass III SnRK2 members in maize (*ZmSnRK2.8/2.9/2.10/2.12*), which, despite high sequence similarity, exhibit differential spatiotemporal distribution. Notably, *ZmSnRK2.8* and *ZmSnRK2.10* are highly expressed in developing endosperm and embryo, indicating their possible involvement in kernel development (Long et al. 2021). However, the precise roles and mechanisms of these kinases in kernel development are still unclear.

In this study, we generated knockout mutants for all four subclass III SnRK2s via CRISPR‐Cas9, demonstrating that these kinases function redundantly to regulate maize kernel development. We further revealed that subclass III SnRK2s mediate sucrose signaling to regulate endosperm starch and zein synthesis through distinct mechanisms, while also mediating ABA signaling to control GLB synthesis and late embryo development via a conserved “ABA–SnRK2–bZIP” pathway. Moreover, overexpression of *ZmSnRK2.10* significantly increased kernel starch and protein content as well as vitreous endosperm (VE) ratio, thereby simultaneously promoting grain yield and quality. Thus, our study deciphers a novel regulatory network involving subclass III SnRK2s that controls kernel storage reserve accumulation and provides valuable genetic resources for maize breeding.

## 2 Results

### 2.1 Subclass III SnRK2s Function Redundantly in Maize Kernel Development and Filling

**FIGURE 1:**
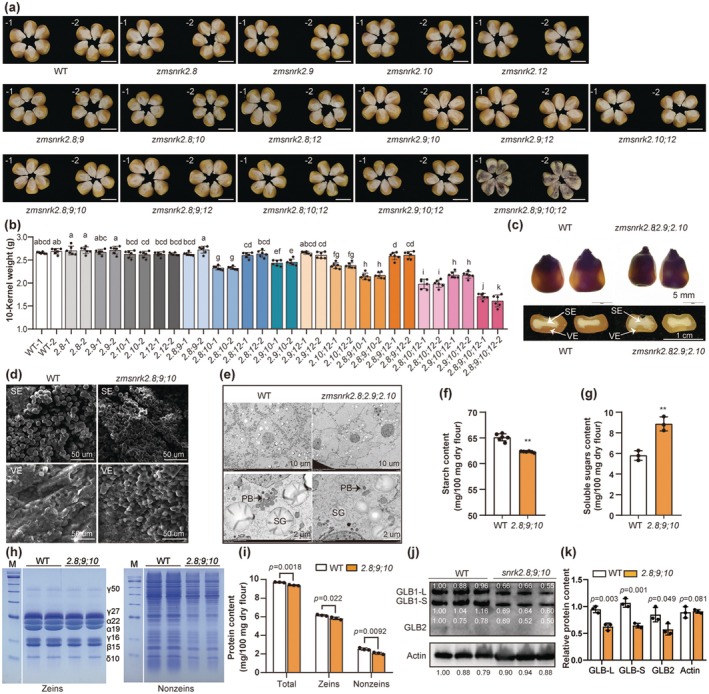
Phenotypic and biochemical analyses of *zmsnrk2s* kernels. (a) The kernel phenotypes of wild‐type (WT) and 15 different genotypes of *zmsnrk2s* knockout mutants. Scale bar, 1 cm. (b) The weight of 10 mature kernels. Data are means ± SD (*n* = 6; different letters indicate significant differences; *p* < 0.05, Duncan multiple range test for each interval). (c) Phenotypic comparison in WT and *zmsnrk2.8;9;10* mature kernels. Top panel, WT and *zmsnrk2.8;9;10* mature kernels observed on a lightbox. Scale bar, 5 mm; Bottom panel, horizontal sections of mature WT and *zmsnrk2.8;9;10* kernels. Scale bar, 1 cm. SE: Starchy endosperm; VE: Vitreous endosperm. (d) SEM images of starch granules in the mature kernels. Scale bar, 50 μm. (e) TEM images of 15 DAP (days after pollination) kernels of WT and *zmsnrk2.8;9;10*. SG: Starch granule; PB: Protein body. Scale bar, 10 μm (up), 2 μm (down). (f) The starch content of WT and *zmsnrk2.8;9;10* mature kernels. Data are means ± SD (*n* = 6). (g) The soluble sugars content of WT and *zmsnrk2.8;9;10* mature kernels. Data are means ± SD (*n* = 3). (h) SDS‐PAGE analysis of zeins (left) and nonzeins (right) accumulations in WT and *zmsnrk2.8;9;10* mature kernels. M, marker; γ50, 50 kD γ‐zein; γ27, 27‐kD γ‐zein; α22, 22‐kD α‐zein; α19, 19‐kD α‐zein; γ16, 16‐kD γ‐zein; β15, 15‐kD β‐zein; δ10, 10‐kD δ‐zein. The marker and sample lanes originated from the same gel but were rearranged for clarity. (i) The protein content (including total protein, zeins, and nonzeins) of WT and *zmsnrk2.8;9;10* mature kernels. Data are means ± SD (*n* = 3). (j) Immunoblot analysis for globulins. (k) The quantitative protein accumulation for globulins according to (j). Actin was used as an internal control. Three independent samples for each material were performed, and the protein bands were quantified using the ImageJ software. Statistical significance (*p < 0.05; **p < 0.01) was determined by two‐tailed Student's *t*‐test, as shown in (f, g, i, k).

To investigate the biological functions of subclass III SnRK2s in maize kernel development, we generated two independent knockout lines for each gene using CRISPR/Cas9 technology, introducing different frameshift mutations in the KN5585 background (Figure S1). All single mutants exhibited no obvious differences in kernel size or weight compared with wild‐type (WT) (Figure 1a,b, Figures S2a and S3a). We then constructed double, triple, and quadruple mutants with all possible combinations through genetic crosses (Figure 1a and Figure S2a,b). Immunoblot analysis indicated that each subclass III SnRK2 was scarcely detected in the corresponding single, double, and triple mutants (Figure S2c). All double mutants with the mutated *ZmSnRK2.10*, including *zmsnrk2.8;2.10*, *zmsnrk2.9;2.10*, and *zmsnrk2.10;2.12*, exhibited significantly reduced kernel size and weight, and more severe defects were observed in triple (*zmsnrk2.8;2.9;2.10, zmsnrk2.8;2.10;2.12*, and *zmsnrk2.9;2.10;2.12*) and quadruple (*zmsnrk2.8;2.9;2.10;2.12*) mutants compared with WT. However, the *zmsnrk2.8;2.9;2.12* mutant kernels did not show any visible phenotype (Figure 1a,b, Figures S2a and S3a). Notably, kernels with homozygous mutations in two or more *ZmSnRK2s* (including *ZmSnRK2.10*) were much smaller than the WT kernels from the same heterozygous ear (Figure S2b), suggesting that defective kernel development in these mutants was primarily caused by filial tissues. Notably, dry‐weight‐based analysis revealed a slight reduction in starch content specifically in *zmsnrk2.10* mutant kernels, whereas protein content remained unchanged in all single‐mutant lines (Figure S3b,c). Consistent with this, gene expression analysis revealed that all four subclass III *ZmSnRK2* genes are expressed during kernel development, with *ZmSnRK2.10* exhibiting the highest transcript levels among them, particularly after 9 days after pollination (DAP) (Figure S4). Together, these findings establish that the four subclass III SnRK2s redundantly regulate maize kernel development, with ZmSnRK2.10 playing a predominant role.

Given the severe vivipary observed in the *zmsnrk2.8;2.9;2.10;2.12* quadruple mutant kernels (Figure 1a), we focused on triple mutants exhibiting pronounced kernel‐filling defects. Considering the high sequence homology between ZmSnRK2.9 and ZmSnRK2.10 (Figure S1), along with the higher expression level of *ZmSnRK2.8* compared with its closest paralog, *ZmSnRK2.12*, in developing kernels (Figure S4), we prioritized the *zmsnrk2.8;2.9;2.10* triple mutant for further analysis. Dynamic analysis of dry matter accumulation showed that the dry weight of mutant kernels is significantly lower than that of the WT from early grain filling (15 DAP) until maturity (Figure S5). Mature mutant kernels exhibited reduced size and diminished vitreous endosperm compared with WT, with smaller starch granules (SGs) embedded in a protein‐depleted matrix (Figure 1c,d). Longitudinal sections of developing kernels (10–15 DAP) further revealed significantly smaller endosperm and embryo in mutants, with endosperm tissue showing a marked reduction in SG density relative to WT (Figure S6). Transmission electron microscopy (TEM) corroborated these observations, confirming fewer and smaller SGs, along with a pronounced decrease in protein body (PB) number in mutant endosperm at 15 DAP (Figure 1e). Additionally, nontargeted metabolomics analysis revealed that sucrose levels, which serve as both substrate and energy for storage compound synthesis, did not differ significantly between *zmsnrk2.8;9;10* mutants and WT kernels at 15 DAP. However, the contents of intermediate metabolites, including glucose and fructose, were significantly elevated in the mutant kernels (Figure S7, Table S1). Correspondingly, mature mutant kernels exhibited significantly reduced starch content and increased soluble sugar content (Figure 1f,g). SDS‐PAGE and biochemical analyses indicated a drastic decrease in total protein, zeins, and nonzeins in these kernels (Figure 1h,i). Additionally, immunoblot analysis revealed a significant reduction in the accumulation of globulins (GLBs), which are the major storage proteins in the embryo (Figure 1j,k). Consequently, the impaired synthesis of starch and storage proteins largely led to a 22% reduction in kernel weight in the *zmsnrk2.8;2.9;2.10* mutants relative to WT (Figure 1b). In summary, these findings demonstrate that subclass III SnRK2s control both starch and storage protein synthesis, thereby influencing maize kernel filling.

### 2.2 Integrated Omics Reveals Subclass III SnRK2s Regulate Starch and Storage Protein Synthesis via Distinct Mechanisms

**FIGURE 2:**
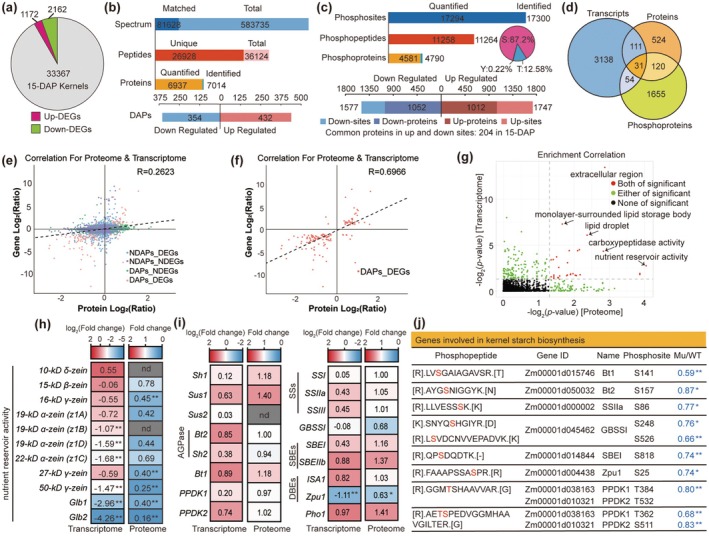
Integrated omics analyses reveal distinct regulatory pathways for starch and storage protein synthesis. (a) The differentially expressed genes (DEGs) in z*msnrk2.8;9;10* compared with the WT from the transcriptome. DEGs were identified based on |fold‐change| ≥ 2 and *p*‐value ≤ 0.05. (b) The sampled spectrum, peptides, proteins, and differentially abundant proteins (DAPs) identified from the proteome. DAPs were identified based on fold‐change ≥ 1.2 or ≤ 0.83, and *p*‐value ≤ 0.05. (c) The sampled phosphosities, phosphopetides, phosphoproteins, and differentially phosphorylated proteins (DPPs) identified from the quantitative phosphoproteome. (d) Venn diagram showing the overlap of DEGs, DAPs, and DPPs. (e) Correlation scatterplot of expression levels for quantitative proteins and their associated genes. The horizontal axis shows the log_2_ expression levels of proteins in each alignment group, and the vertical axis shows the log_2_ expression levels of corresponding genes. (f) Correlation scatterplot of DAPs and DEGs. (g) Scatterplot of gene ontology (GO) enrichment correlation of transcriptome and proteome. (h, i) Heatmap of zein and globulin genes (h) and key starch synthesis‐related genes (i) at both mRNA and protein levels. nd, not identified. Items with corrected *p* < 0.05 were considered as DEGs or DAPs, and the *p‐*values are indicated with stars (*p < 0.05; **p < 0.01). (j) List of key starch synthesis‐related enzymes from phosphoproteome, with phosphopeptide(s), gene ID, phosphosites, and the changes in phosphorylation levels in *zmsnrk2.8;9;10* compared with WT.

To understand how subclass III SnRK2s control kernel filling, we conducted multi‐omics profiling of 15‐DAP kernels from *zmsnrk2.8;2.9;2.10* and WT, using three independent biological replicates (Figure S8a–c). Deep transcriptome sequencing revealed 3334 differentially expressed genes (DEGs) in the mutant compared with WT, with 1172 upregulated and 2162 downregulated (fold change ≥ 2 and *p* ≤ 0.05) (Figure 2a, Figure S8d and Table S2). We further performed quantitative proteomics and phosphoproteomics analyses using liquid chromatography–tandem mass spectrometry (LC/MS–MS) on tandem mass tag (TMT)‐labeled peptides, and detected 786 differentially abundant proteins (DAPs) among 6937 quantified proteins (432 up and 354 down, fold change ≥ 1.2 or ≤ 0.83 and *p* ≤ 0.05) (Figure 2b, Figure S8e and Table S3). Additionally, we identified a total of 17294 phosphosites across 4581 quantified phosphoproteins. Of these, 1747 phosphosites in 1012 phosphoproteins displayed enhanced phosphorylation, while 1577 phosphosites in 1052 phosphoproteins had reduced phosphorylation in the mutant (fold change ≥ 1.2 or ≤ 0.83 and *p* ≤ 0.05). Notably, 204 proteins showed both enhanced and reduced phosphorylation at different sites (Figure 2c, Figure S8f and Table S4). Motif enrichment analysis using MEME identified the two most significant motifs, LxRSxS and RSxSP, in differentially phosphorylated proteins (DPPs) (Figure S9a,b). The LxRSxS motif is known to be phosphorylated at the serine residue by subclass III SnRK2s (Furihata et al. 2006). Structural domain classification revealed an enrichment of differentially phosphorylated proteins, including protein kinases, protein phosphatases 2C (PP2C), and transcription factors (TFs) (e.g., bZIP, Myb, bHLH) (Figure S9c). This is consistent with the established roles of PP2C as upstream regulators of SnRK2s and bZIP TFs as key SnRK2 targets (Fujii and Zhu 2009; Wang et al. 2013). Gene Ontology (GO) and Kyoto Encyclopedia of Genes and Genomes (KEGG) analyses of DPPs revealed significant enrichment in pathways related to starch and sucrose metabolism, glycolysis, transcription regulator activity, transporter activity, and plant hormone signal transduction (Figure S9d,e).

Integrated analysis of three omics data revealed that 18.1% of DAPs (142 out of 786) had corresponding DEGs in *zmsnrk2.8;2.9;2.10*, most of which (133 out of 142) followed the same expression trend as their transcripts (Table S5). In contrast, only 4.6% and 8.1% of DPPs (85 and 151 out of 1860) displayed changes in their cognate transcripts and proteins, respectively (Figure 2d, Figure S10a and Table S5). This indicates that DPPs are primarily regulated by posttranslational modification, consistent with SnRK2 kinases acting mainly through substrate phosphorylation. Additionally, there was a weak correlation between the transcriptome and proteome in *zmsnrk2.8;2.9;2.10* (*R* = 0.2632) (Figure 2e). However, a strong positive correlation was observed between DEGs and DAPs (*R* = 0.6966), with an even higher correlation for those sharing the same trend (*R* = 0.7201) (Figure 2f and Figure S10b,c). GO enrichment and correlation analyses for both the proteome and transcriptome revealed shared significant terms, including nutrient reservoir activity, carboxypeptidase activity, extracellular region, monolayer‐surrounded lipid storage body, and lipid droplet (Figure 2g). Many genes encoding storage proteins, such as zeins and GLBs, were enriched in the nutrient reservoir activity category and displayed decreased transcript levels and protein abundance in *zmsnrk2.8;2.9;2.10* compared with WT (Figure 2h and Figure S11a,b), consistent with reduced zein and non‐zein contents observed in the mutant (Figure 1h–k), suggesting that subclass III SnRK2s regulate storage protein synthesis in maize at the transcriptional level.

Despite the significant reduction in starch content in the *zmsnrk2.8;2.9;2.10* kernels (Figure 1f), core starch synthesis‐related genes (SSRGs) in maize endosperm exhibited no significant changes at the transcript or protein levels (Figure 2i). Quantitative real‐time PCR (qRT‐PCR) and immunoblot analyses confirmed these findings (Figure S11c–e). However, the activities of key starch synthesis‐related enzymes (SSREs), including AGPase, SSs, SBEs, and DBEs, were significantly reduced in the mutant kernels (Figure S12). We therefore investigated the phosphorylation status of these SSREs, finding that most contained multiple phosphosites (Figure S13). Notably, several phosphosites in Bt1, Bt2, SSIII, GBSSI, SBEI, and Zpu1 exhibited significantly reduced phosphorylation in the mutant (Figure 2j). These results suggest that subclass III SnRK2s regulate endosperm starch synthesis, probably by modulating SSRE activities via phosphorylation.

### 2.3 Phosphorylation of Bt1 by Subclass III SnRK2s Enhances Its Activity and Promotes Starch Synthesis

**FIGURE 3:**
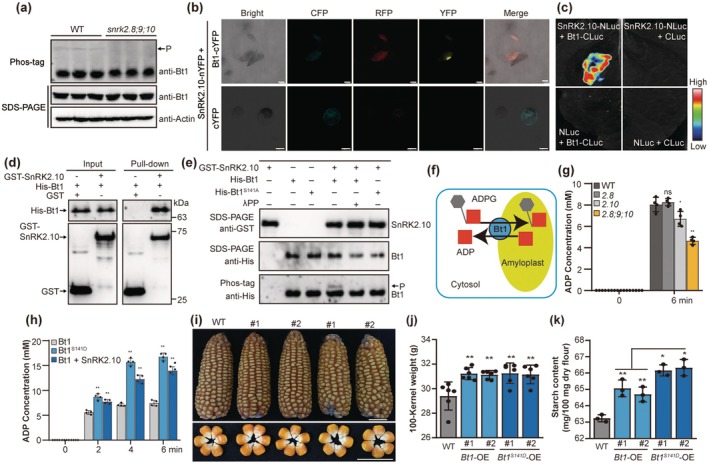
Direct phosphorylation of Bt1 by ZmSnRK2.10 enhances ADP‐glucose transport and starch synthesis. (a) Bt1 protein abundance and the phosphorylation levels in z*msnrk2.8;9;10* and WT kernels at 15‐DAP. The slow migrated band indicated the phosphorylated Bt1 protein in the Phos‐tag gel and is marked with the arrow. Actin was used as an internal control. (b) BiFC assay showing the interaction between ZmSnRK2.10 with Bt1 in maize leaf protoplasts. Scale bars, 10 μm. CFP, cyan fluorescent protein. YFP, yellow fluorescent protein. (c) LCI assay showing the interaction between ZmSnRK2.10 with Bt1 in *Nicotiana benthamiana* leaves. High, strong LUC intensity; Low, weak LUC intensity. (d) GST pull‐down assay showing the interaction between ZmSnRK2.10 with Bt1. (e) In vitro kinase assay showing phosphorylation modification of Bt1 by ZmSnRK2.10 and identification of phosphorylation sites. Lambda protein phosphatase (λPP) was used to dephosphorylate the induced phosphorylation proteins. The slow migrated band indicated the phosphorylated Bt1 protein in the Phos‐tag gel and is marked with the arrow. (f) Schematic diagram of Bt1‐mediated transport of ADP‐Glc (ADPG) into amyloplasts. (g) Measurement of ADP efflux from purified amyloplasts in z*msnrk2.8;9;10* and WT kernels at 15‐DAP. (h) Bt1 phosphorylation by ZmSnRK2.10 enhances its ADPG transport capacity in purified amyloplasts. (i) The ear and kernel phenotypes of the WT, *Bt1*‐overexpression (*Bt1*‐OE), and *Bt1* ^*S141D*^‐overexpression (*Bt1* ^*S141D*^‐OE) lines. Scale bar, 3 cm. (j) 100‐kernel weight of mature WT, *Bt1*‐OE, and *Bt1* ^*S141D*^‐OE. Data are means ± SD (*n* = 6). (k) The starch content of mature WT, *Bt1*‐OE, and *Bt1* ^*S141D*^‐OE kernels. Data are means ± SD (*n* = 3). Statistical significance (*p < 0.05; **p < 0.01) was determined by two‐tailed Student's *t*‐test, as shown in (g, h, j, k).

To investigate the effects of subclass III SnRK2s on SSRE phosphorylation and activity, we conducted an immunoprecipitation mass spectrometry (IP–MS) assay using an anti‐ZmSnRK2.10 antibody. This analysis identified several endosperm‐specific SSREs that co‐precipitated with ZmSnRK2.10, including Bt1, Bt2, GBSSI, SSIII, SBEI, and Zpu1 (Figure S14a), all of which harbored sites with decreased phosphorylation in the *zmsnrk2.8;2.9;2.10* kernels (Figure 2j). Consistent with these findings, AI–PPI prediction indicated direct interactions between ZmSnRK2.10 and these SSREs (Figure S14b). Given that Bt1 exhibited the greatest reduction in phosphorylation abundance and the strongest predicted interaction with ZmSnRK2.10 (score = 0.9406) (Figure 2j and Figure S14b), we focused subsequent analysis on the regulatory role of subclass III SnRK2s on Bt1. We first confirmed significantly reduced phosphorylation of Bt1 in *zmsnrk2.8;2.9;2.10* kernels using Phos‐tag SDS‐PAGE (Figure 3a). Bt1 is a plastid membrane protein, while subclass III SnRK2s localize to the cytosol and nucleus (Long et al. 2021). However, immunofluorescence revealed cytosol‐localized ZmSnRK2.10 adjacent to plastids and proximal to Bt1 in endosperm cells (Figure S15). Furthermore, bimolecular fluorescence complementation (BiFC) assays showed that both ZmSnRK2.10 and its homolog ZmSnRK2.8 directly interact with Bt1 around the plastid (Figure 3b and Figure S16a). These results indicate that their interaction occurs at the plastid periphery. Luciferase complementation imaging (LCI) and GST pull‐down assays further confirmed that ZmSnRK2.10 and ZmSnRK2.8 directly interact with Bt1 in vitro and in vivo (Figure 3c,d and Figure S16b–d). Moreover, LC–MS/MS analysis revealed that phosphorylation of Ser141 in Bt1, which resides within a canonical SnRK2 recognition motif (R‐X‐X‐S), depends on subclass III SnRK2 kinases (Figure S16e). This suggests subclass III SnRK2s directly phosphorylate Bt1 at Ser141. To verify this, we performed in vitro phosphorylation assays, demonstrating that ZmSnRK2.8 and ZmSnRK2.10 phosphorylated Bt1, but not the phosphoablative mutant Bt1^S141A^ (Figure 3e and Figure S16f). These results suggest that subclass III SnRK2s physically interact with and phosphorylate Bt1 at Ser141 in developing endosperm.

Bt1 is a member of the mitochondrial carrier family (MCF), characterized by six conserved transmembrane α‐helices (H1–H6) (Pebay‐Peyroula et al. 2003; Kirchberger et al. 2007) (Figure S17a). Mutations at phosphorylation sites within the α‐helices of the yeast MCF protein ANT1 abolished the transport activity (Feng et al. 2010). Ser141, a conserved phosphorylation site located in the first α‐helix (H1) of Bt1, undergoes phosphorylation that induces conformational changes in Bt1 (Figure S17b,c). This suggests subclass III SnRK2‐mediated phosphorylation of Ser141 may regulate Bt1 transporter activity. Bt1 was reported to facilitate the transport of ADPG across the amyloplast envelope in counterexchange with ADP (Figure 3f) (Kirchberger et al. 2007). To further assess Bt1 transport activity, we measured the concentration of ADP released from amyloplasts in both WT and *zmsnrk2.8;2.9;2.10*. The results demonstrated a significant reduction in Bt1 transport activity in the mutant compared with WT (Figure 3g). Incubation of purified Bt1 with intact amyloplasts revealed a time‐dependent increase in ADP efflux, with a more pronounced increase observed when using Bt1^S141D^ (a phosphomimetic Ser‐to‐Asp mutant) or Bt1 in conjunction with ZmSnRK2.10, indicating that ZmSnRK2.10 phosphorylates Bt1, thereby enhancing its transport activity (Figure 3h).

To further investigate the role of Bt1 phosphorylation in starch synthesis, we overexpressed *Bt1* and *Bt1* ^*S141D*^ in maize (Figure S18). Both overexpression lines showed significantly increased kernel size, 100‐kernel weight, and starch content compared with WT (Figure 3i–k). Notably, kernels from the *Bt1* ^*S141D*^ overexpression lines displayed greater kernel size and starch content than those from the *Bt1* overexpression lines, indicating that Bt1 phosphorylation promotes endosperm starch synthesis (Figure 3i–k). However, no significant difference in 100‐kernel weight was observed between *Bt1* and *Bt1* ^*S141D*^ overexpression lines (Figure 3j), potentially due to a trade‐off between starch and other storage substances under limited photoassimilate supply. Collectively, these findings confirm that subclass III SnRK2s regulate starch synthesis in maize endosperm by modulating the activities of Bt1 through phosphorylation.

Given that multiple SSREs were detected to interact with ZmSnRK2.10 in both the IP–MS assay and AI–PPI (Figure S14), we next examined whether subclass III SnRK2s also regulate the phosphorylation of other core SSREs. To this end, we performed Phos‐tag SDS‐PAGE analysis. Consistent with our phosphoproteomic data, the phosphorylation levels of Bt2, PPDK, Zpu1, and GBSSI were significantly reduced in the *zmsnrk2.8;2.9;2.10* kernels (Figure S19a). Furthermore, interaction assays demonstrated that ZmSnRK2.10 interacts with PPDK2 and GBSSI, but not with Bt2 or SBE1 (Figure S19b,c). However, due to their exceptionally large molecular sizes, we were unable to generate amplifiable constructs for SSIII and Zpu1 to assess interactions. Notably, several core SSREs (likely SSs, SBEs, and DBEs) and PPDKs typically assemble into heteromeric complexes within the amyloplast (Hennen‐Bierwagen et al. 2009). Immunofluorescence analysis revealed that these amyloplast‐localized proteins, such as PPDK2, GBSSI, and Zpu1, were predominantly localized in the amyloplast but were also detectable in the cytoplasm. Notably, their cytoplasmic distribution partially overlapped with that of ZmSnRK2.10 (Figure S19d). Collectively, these findings suggest that subclass III SnRK2s directly or indirectly regulate the phosphorylation of these core SSREs, and this regulation likely occurs on the cytoplasmic face of the amyloplast envelope before their import.

### 2.4 Subclass III SnRK2s Regulate Storage Protein Synthesis by Modulating TF Phosphorylation

**FIGURE 4:**
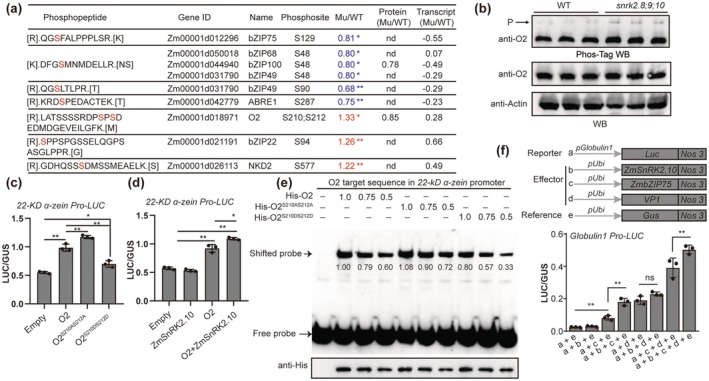
Subclass III SnRK2s regulate zein and globulin synthesis by modulating TF phosphorylation. (a) List of key TFs related to kernel filling and development from subclass III ZmSnRK2s dependent phosphoproteins, including gene IDs, phosphopeptide, phosphosites, and phosphorylation changes in transcription and protein levels in z*msnrk2.8;9;10* compared with WT. (b) O2 protein abundance and the phosphorylation levels in z*msnrk2.8;9;10* and WT kernels at 15‐DAP. The slow migrated band indicated the phosphorylated O2 protein in the Phos‐tag gel and is marked with the arrow. Actin was used as an internal control. (c) Altered transactivation capacity of phosphorylation‐mimetic (O2^S210D212D^) and dephosphorylated (O2^S210A212A^) forms of O2 on 22‐KD *α‐zein* promoter. (d) ZmSnRK2.10 enhanced the transactivation capacity of O2 on 22‐KD *α‐zein* promoter. Data are means ± SD (*n* = 3). (e) EMSA assays showing the binding affinities of O2, O2^S210A212A^, and O2^S210D212D^ to the 22‐KD *α‐zein* promoter at decreasing protein concentrations. The shifted probe level was evaluated using the ImageJ software. (f) ZmSnRK2.10 enhanced the transactivation activity of ZmbZIP75 on the *Globulin1* promoter. The LUC/GUS ratios were tested in the maize endosperm (c, d) or embryo (f) cells through co‐bombardment with the reporter and effector constructs. Data are means ± SD (*n* = 3). Statistical significance (*p < 0.05; **p < 0.01) was determined by two‐tailed Student's *t*‐test, as shown in (c, d, f).

Since subclass III SnRK2s regulate the synthesis of storage proteins such as zeins and GLBs at the transcriptional level (Figure 2h), we hypothesized that specific TFs downstream of these SnRK2s mediate this regulation. An integrated omics analysis identified 103 out of 1290 kernel‐expressed TFs with differential phosphorylation. Notably, most of these TFs (98 out of 103) exhibited no significant changes in transcript or protein levels in the *zmsnrk2.8;2.9;2.10* mutant (Figure S20 and Table S6), indicating strong phosphorylation modifications. Among these, several TFs, including ZmbZIP49, ZmbZIP68, ZmbZIP75, and ZmbZIP100, belong to the A‐Group bZIP family, which are recognized as direct targets for subclass III SnRK2s in mediating ABA responses (Dröge‐Laser et al. 2018). Additionally, several known TFs associated with kernel development, such as Opaque2 (O2), bZIP22, and NKD2, displayed hyperphosphorylation in the mutant (Figure 4a).

O2 is a central TF for zein synthesis in maize endosperm, and its hyperphosphorylation inhibits transcriptional activity (Ciceri et al. 1997). The two adjacent serine residues (Ser210 and Ser212) within the O2 protein serve as the predominant phosphorylation sites in vivo (Walley et al. 2013), exhibiting significantly enhanced phosphorylation in *zmsnrk2.8;2.9;2.10* (Figure 4a and Figure S21a). Moreover, BiFC and LCI assays revealed no direct interaction between either ZmSnRK2.10 or ZmSnRK2.8 and O2 (Figure S21b,c). The observed hyperphosphorylation of O2 in *zmsnrk2.8;9;10* kernels likely results from subclass III SnRK2‐mediated inhibition of kinases targeting Ser210/Ser212 (e.g., GSK11, CDK2, and CDK10), as suggested by NetPhos3 predictions and multi‐omics data (Figure S21d,e and Table S7). To assess the regulatory effects of phosphorylation at these sites on zein gene expression, we created two O2 variants: O2^S210A/S212A^ and O2^S210D/S212D^ to mimic the phosphorylation and nonphosphorylation, respectively. These variants were driven by the *ubiquitin* promoter as effectors, while the 22‐kD *α‐zein* promoter driving the *LUC* gene served as a reporter. Transient expression assays showed that O2^S210A/S212A^ significantly enhanced 22‐kD *α‐zein* promoter activity compared with native O2, while O2^S210D/S212D^ diminished this effect (Figure 4c). Moreover, the transactivation of the 22‐kD *α‐zein* promoter by O2 was significantly enhanced in the presence of ZmSnRK2.10 (Figure 4d). Moreover, recombinant proteins His‐O2, His‐O2^S210A/S212A^, and His‐O2^S210D/S212D^ were successfully expressed and purified, respectively (Figure S22). Electrophoretic mobility shift assay (EMSA) analysis indicated that phosphorylation at Ser210 and Ser212 impaired the DNA binding ability of O2, while dephosphorylation enhanced it (Figure 4e), consistent with a previous report (Ciceri et al. 1997). However, both yeast transcriptional activation assays and dual‐luciferase reporter systems demonstrated that the phosphorylation of O2 at S210/S212 does not affect its transactivation capability (Figure S23). These results suggest that subclass III SnRK2s enhance O2 activity through indirectly sustaining its hypophosphorylation, thereby promoting endosperm zein synthesis.

In maize, globulin proteins predominantly accumulate in the embryo during late‐stage kernel development, with their corresponding genes primarily regulated through the synergistic interaction of TF VP1 and ABA (Rivin and Grudt 1991). VP1 requires additional ABA‐responsive TFs that bind to the ABRE element to regulate *Glb1* transcription (Zheng et al. 2019). ZmbZIP75, homologous to Arabidopsis ABI5, was shown to interact with VP1 to regulate downstream genes (Zhang, Sun, et al. 2019). In the *zmsnrk2.8;2.9;2.10* mutant, the phosphorylation of ZmbZIP75 at Ser129 was significantly decreased. (Figure 4a). Consistent with our previous findings, we demonstrate that ZmSnRK2.10 directly phosphorylates ZmbZIP75 at Ser129, thereby enhancing its protein stability to regulate late‐stage kernel development through ABA signaling (Long et al. 2024). Notably, the greatest enhancement of *Glb1* promoter activity was observed with the co‐transformation of ZmSnRK2.10, VP1, and ZmbZIP75 (Figure 4f). These results suggest that ZmbZIP75 phosphorylation by ZmSnRK2.10 and its interaction with VP1 are essential for GLB synthesis. In summary, these findings demonstrate that ZmSnRK2.10 regulates storage protein synthesis in both the endosperm and the embryo by modulating specific TF phosphorylation.

### 2.5 ZmSnRK2.10 is Sequentially Activated by Sucrose and ABA During Kernel Development

**FIGURE 5:**
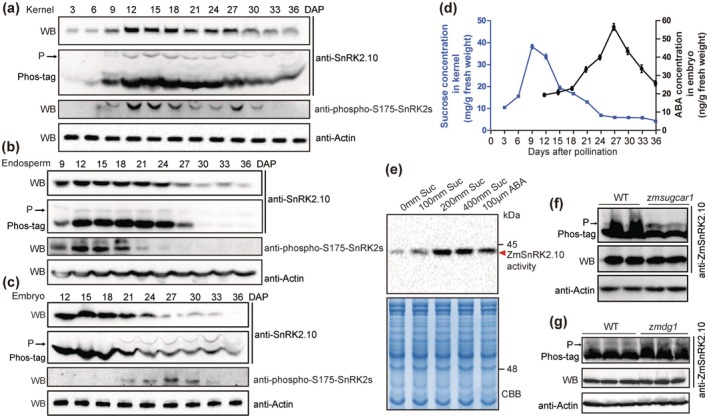
ZmSnRK2.10 is sequentially activated by sucrose and ABA during kernel development. (a–c) ZmSnRK2.10 protein abundance and the phosphorylation levels in developing kernels (a), endosperms (b) and embryos (c). The slow migrated band indicated the phosphorylated ZmSnRK2.10 protein in the Phos‐tag gel and is marked with the arrow. SnRK2 autophosphorylation activity was detected with anti‐phospho‐S175‐SnRK2s (a–c). Actin was used as an internal control. (d) Concentrations of sucrose and ABA in developing maize kernels. Data are means ± SD (*n* = 3). (e) In‐gel kinase assay of ZmSnRK2.10 under sucrose and ABA treatment. Total proteins prepared from 15‐DAP kernels treated with sucrose or ABA for 1 h were incubated with anti‐ZmSnRK2.10 agarose beads and separated by SDS‐PAGE gel containing 0.3 mg/mL MBP substrate. ZmSnRK2.10 kinase activity was detected by autoradiography. The red arrow indicates the target protein bands. Coomassie Brilliant Blue (CBB) staining provided a loading control. (f, g) ZmSnRK2.10 protein abundance and the phosphorylation levels in 15‐DAP kernels of *zmsugcar1* (f) and *zmdg1* (g). The slow migrated band indicated the phosphorylated ZmSnRK2.10 protein in the Phos‐tag gel and is marked with the arrow. Actin was used as an internal control.

Previous studies have indicated that the activation of subclass III SnRK2s requires autophosphorylation or phosphorylation by B3 Raf‐like kinases (Lin et al. 2021; Saruhashi et al. 2015). To investigate the dynamics of ZmSnRK2.10 protein accumulation and phosphorylation during maize kernel development, we performed immunoblot and Phos‐tag SDS‐PAGE analyses using an anti‐ZmSnRK2.10 antibody. To further assess subclass III SnRK2s autophosphorylation activity during this developmental period, we measured activity levels using an anti‐phospho‐S175‐SnRK2 antibody (Zhao et al. 2018). These analyses revealed that ZmSnRK2.10 predominantly accumulates in developing kernels during the critical kernel‐filling stage (9–27 DAP) (Figure 5a and Figure S24), consistent with qRT‐PCR results (Figure S4). Combined immunoblot and immunofluorescence analyses demonstrated that ZmSnRK2.10 protein maintains sustained abundance in both the endosperm and embryo throughout this critical phase (Figure 5b,c and Figure S25a). Furthermore, ZmSnRK2.10 exhibits dual cytoplasmic and nuclear localization in the endosperm, while demonstrating predominant nuclear localization in the embryo, particularly during late kernel development (Figure S25b). Notably, ZmSnRK2.10 phosphorylation was first detectable at 9 DAP and sustained until 33 DAP in developing kernels, with particularly high levels observed from 12 to 27 DAP. The autophosphorylation activity of subclass III SnRK2s paralleled the accumulation of phosphorylated ZmSnRK2.10 (Figure 5a). Significantly, high phosphorylation levels and autophosphorylation activity were observed in the endosperm during early kernel filling (9–27 DAP) (Figure 5b). In contrast, the embryo exhibited sustained phosphorylation and autophosphorylation activity during late kernel development (21–33 DAP), despite a decrease in ZmSnRK2.10 protein levels at late stages (27–36 DAP) (Figure 5c). This temporal and spatial expression pattern implies that ZmSnRK2.10 may transduce distinct signaling cascades to orchestrate differential regulatory mechanisms governing physiological functions in these developing tissues.

The activation of subclass III SnRK2s is known to rely on high ABA concentrations (Cutler et al. 2010). Studies indicate that ABA maintains basal levels in the endosperm throughout kernel filling, whereas embryos undergo substantial ABA accumulation during late development (Jones and Brenner 1987). These findings suggest that other signals might activate ZmSnRK2.10 during endosperm filling. Sucrose, a key signaling molecule for endosperm filling (Li et al. 2020; Yang et al. 2024), maintained high levels during the early kernel filling phase (9–18 DAP) (Figure 5d), correlating with ZmSnRK2.10 phosphorylation and autophosphorylation activity in the endosperm (Figure 5b). Moreover, the ABA concentration of the embryo gradually increased after 18 DAP, peaking at 27 DAP and then decreasing again towards the end of development (Figure 5d), correlating with ZmSnRK2.10 phosphorylation and autophosphorylation activity in the embryo (Figure 5c). Additionally, we analyzed sucrose and ABA contents separately in the embryo and endosperm at 12‐ and 27‐DAP (Figure S26a,b). The results further clarify the correlation between the ZmSnRK2.10 activity and endospermic sucrose or embryonic ABA. In‐gel kinase assays on 15‐DAP endosperm and embryo treated with sucrose and ABA showed that sucrose notably increased ZmSnRK2 activities in the endosperm, while ABA treatment resulted in a marginal increase. Conversely, in the embryo, ABA significantly enhanced ZmSnRK2 activities, with a minor effect from sucrose (Figure S26c). To further investigate the effects of sucrose on ZmSnRK2.10 activity, 15‐DAP kernels were treated with varying sucrose concentrations and then analysed using Phos‐tag SDS‐PAGE and in vitro kinase assays. The results showed that ZmSnRK2.10 activity and phosphorylation levels increased in a sucrose concentration‐dependent manner (Figure 5e and Figure S26d).

To assess whether sucrose plays a dominant role in ZmSnRK2.10 activation during endosperm filling, we analyzed ZmSnRK2.10 phosphorylation levels in 15‐DAP kernels of maize *zmsugcar1* mutant, which has severely reduced sucrose content due to impaired sugar transport (Yang, Wang, Yu, et al. 2022). Interestingly, ABA levels in the *zmsugcar1* kernels were similar to those in WT (Figure S27). Immunoblot analyses indicated no significant difference in ZmSnRK2.10 protein levels between the mutant and WT, but phosphorylation levels were drastically reduced in the mutant kernels (Figure 5f). In contrast, the *dg1* mutant, with defective ABA accumulation but increased sucrose in kernels (Qin et al. 2021), showed higher ZmSnRK2.10 phosphorylation compared with WT, despite similar protein abundance (Figure 5g). Additionally, based on published transcriptome data (Huang et al. 2016), all zein genes downregulated in *zmsnrk2.8;2.9;2.10* were significantly induced by sucrose but repressed by ABA in maize endosperm (Figure S28), suggesting that subclass III SnRK2s regulate zein protein synthesis in maize endosperm independent of ABA, more likely dependent on sucrose. Collectively, these findings demonstrate that ZmSnRK2.10 is sequentially activated by sucrose in the endosperm and ABA in the embryo to orchestrate endosperm filling and late embryo development in maize.

### 2.6 Overexpression of ZmSnRK2.10 Enhances Maize Yield and Quality

**FIGURE 6:**
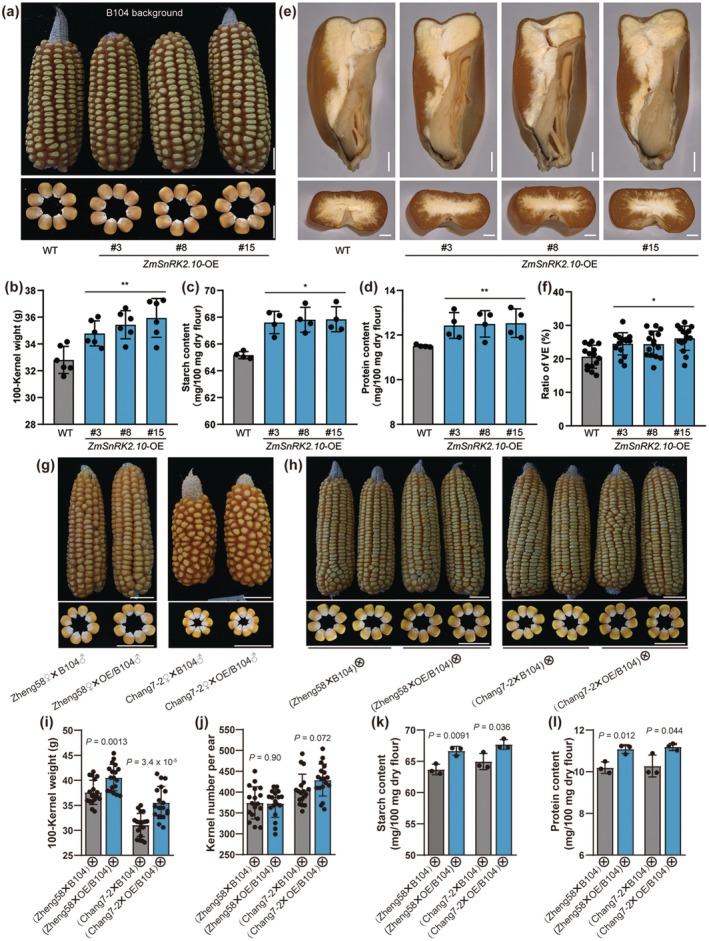
*ZmSnRK2.10* overexpression enhances grain yield and quality. (a) The ear and kernel phenotypes of the WT and *ZmSnRK2.10*‐overexpression (*ZmSnRK2.10*‐OE) lines. (b–d) Measurement of 100‐kernel weight (b, *n* = 6), starch content (c, *n* = 4), and protein content (d, *n* = 4) of the mature WT and *ZmSnRK2.10*‐OE kernels harvested in Sanya. Data are means ± SD. (e) Section observations of mature kernels of WT and *ZmSnRK2.10*‐OE. Upper panel, longitudinal section. Bottom panel, transverse section. Scale bars, 200 μm. (f) Measurement of the ratio of vitreous endosperm (VE). Data are means ± SD (*n* = 15). (g) The ear and kernel phenotypes of Zheng58 × B104, Zheng58 × OE/B104, Chang7‐2 × B104 and Chang7‐2 × OE/B104. The overexpression line OE15 and the background B104 were crossed to two representative inbred lines. (h) The ear and kernel phenotypes of F_1_ hybrids. (i–l) Measurement of the 100‐kernel weight (i, *n* = 18), kernel number per ear (j, *n* = 18), starch content (k, *n* = 3), and protein content (l, *n* = 3) of F_1_ hybrids. Data are means ± SD. Statistical significance (*p < 0.05; **p < 0.01) was determined by two‐tailed Student's *t*‐test, as shown in (b–d, f, i–l).

To assess the potential of ZmSnRK2.10 in breeding practices, we overexpressed *ZmSnRK2.10* in the maize inbred line B104 under the control of the maize *Ubiquitin* promoter (Figure S29a). Three independent overexpression lines (OE‐3, OE‐8, and OE‐15) with significantly elevated ZmSnRK2.10 transcript and protein levels were selected for further analysis (Figure S29b–d). Compared with WT plants, these overexpression lines exhibited notably increased kernel size and 100‐kernel weight in Sanya (Figure 6a,b). At 15‐DAP, the activities of SSREs, including AGPase, SSs, SBEs, and DBEs, were markedly enhanced in the overexpression lines (Figure S29e), resulting in a significant increase in the starch content of mature kernels compared with WT (Figure 6c). Additionally, the protein content and vitreous endosperm (VE) ratio in the overexpressed kernels were also markedly higher than those in WT (Figure 6d–f).

To evaluate the impact of *ZmSnRK2.10* overexpression in hybrids, we crossed the overexpression line OE‐15 (as the paternal parent) with two elite inbred lines: Zheng58 and Chang7‐2, which are parents of the widely cultivated Zhengdan958 hybrid in China. The resulting kernels from the crosses Zheng58♀ × OE/B104♂ and Chang7‐2♀ × OE/B104♂ were significantly larger than those from the corresponding WT crosses (Figure 6g), suggesting that *ZmSnRK2.10* overexpression in filial tissues increases kernel size. Field trials showed that F_1_ hybrids Zheng58 × OE/B104 and Chang7‐2 × OE/B104 exhibited significantly increased kernel width and thickness compared with their WT counterparts (Figure 6h and Figure S30). The 100‐kernel weight of these overexpression hybrids increased by 7.8% and 14.4%, respectively, compared with the WT controls (Figure 6i), while kernel number per ear displayed no significant changes (Figure 6j), indicating a substantial increase in grain yield per plant. Notably, both kernel starch and protein contents were significantly elevated in the *ZmSnRK2.10* overexpression hybrids (Figure 6k,l), consistent with observations in the B104 background (Figure 6c,d). Collectively, these findings reveal that overexpression of *ZmSnRK2.10* in maize enhances both starch and protein accumulation, thereby simultaneously improving grain yield and quality.

## 3 Discussion

### 3.1 Subclass III SnRK2s Orchestrate Maize Kernel Filling, With ZmSnRK2.10 Playing a Predominant Role

Subclass III SnRK2s, a central node of ABA signaling, are widely involved in seed dormancy, germination and various stress responses (Mao et al. 2020). Unexpectedly, they also affect plant growth and development under favorable conditions (Yoshida et al. 2019; Zheng et al. 2010). However, the role of Subclass III SnRK2s in crucial processes such as kernel filling in crops has remained largely elusive. In this study, we demonstrated that subclass III SnRK2s function redundantly, but they are essential for kernel filling and development in maize. Knockout of subclass III *ZmSnRK2s* genes induced severe impairment in kernel filling, characterized by a significant reduction in starch and storage protein accumulation. Moreover, homozygous mutant kernels derived from the same self‐pollinated heterozygous ear displayed marked phenotypic defects, including poor filling and vivipary (Figure S2 and Figure 1). These results clearly demonstrate that subclass III SnRK2s act on filial tissues (endosperm or embryo) to regulate maize kernel filling and development under nonstress conditions.

In Arabidopsis, the *snrk2.2/2.3/2.6* triple mutant shows severe developmental defects, whereas the *snrk2.6* single mutant and *snrk2.2/2.3* double mutant are morphologically similar to the WT under normal conditions. Functional characterization revealed that *SnRK2.6* appeared to predominantly regulate sucrose metabolism and overall plant growth, whereas *SnRK2.2/2.3* specialized in seed germination (Fujii and Zhu 2009; Nakashima et al. 2009; Zheng et al. 2010). In this study, kernels of all single mutants, including *zmsnrk2.10*, exhibited no visible phenotypic alterations (Figure 1a,b). However, biomass‐normalized analysis revealed a slight reduction in starch content exclusively in *zmsnrk2.10* single mutant kernels (Figure S3). Kernel defects became severe only when *ZmSnRK2.10* mutations were combined with those of other subclass III *SnRK2* members, whereas mutants lacking combinations of other subclass III members (*zmsnrk2.8;9;12*) displayed normal kernel phenotypes (Figure 1a,b). These genetic analyses demonstrate general functional redundancy among subclass III ZmSnRK2s during maize kernel development. However, ZmSnRK2.10 plays a critical role in promoting kernel filling, a function that cannot be fully compensated for by other paralogs. Supporting this, *ZmSnRK2.10* transcripts and proteins preferentially accumulated in both the embryo and endosperm throughout kernel‐filling stages, peaking during the rapid filling period (Figure S4 and Figure 5a–c). Thus, although functional redundancy masks phenotypic consequences in most single mutants, *ZmSnRK2.10* emerges as the principal regulator of kernel filling, attributable to its spatiotemporal expression predominance and the inability of other subclass III members to compensate for its absence. This redundancy likely explains why *ZmSnRK2.10* was not identified in the extensive forward genetic screening for kernel‐defect mutants using EMS‐induced or Mu‐induced heritable mutations. Moreover, overexpression of *ZmSnRK2.10* in both inbred and hybrid backgrounds significantly increases the accumulation of starch and protein, and ultimately increases the kernel size and weight (Figure 6). Thus, these findings indicate the positive regulatory role of the kinase ZmSnRK2.10 in maize kernel filling and the potential value of its application in breeding programs aimed at improving yield and quality.

### 3.2 ZmSnRK2.10 Modulates Endosperm Filling Through Coordinated Regulation of Starch and Zein Biosynthesis via Two Different Ways

Starch and zeins are synthesized synchronously in maize endosperm (Keeling and Myers 2010). A recent study identified two duplicate TFs, ZmNAC128 and ZmNAC130, which directly activate the transcription of both core SSRGs and zein genes, thereby promoting endosperm filling (Chen et al. 2023). Phosphorylation plays critical regulatory roles in endosperm filling, mediating spatiotemporal control of reserve substance biosynthesis (Walley et al. 2013; Yang et al. 2023). However, the protein kinase‐substrate network governing endosperm filling remains poorly characterized. Yang, Wang, Guo, et al. (2022) recently demonstrated that the ABA‐induced subclass II SnRK2 member, SnRK2.2, directly phosphorylates the transcription factors (TFs) bZIP29, ABI19, and O2, to enhance their transactivation potential and promote maize endosperm filling. This highlights the critical regulatory role of SnRK2‐mediated TF phosphorylation, particularly of O2, in regulating zein synthesis. Moreover, the detailed functional characterization of SnRK2.2 in maize kernels requires further investigation. In this study, we identify *ZmSnRK2.10* as the predominant subclass III SnRK2 kinase in maize kernels and uncover its dual regulatory roles in endosperm filling: (1) by directly phosphorylating core SSREs (e.g., Bt1) to enhance enzymatic activities in starch biosynthesis, and (2) by indirectly modulating O2 through suppression of its phosphorylation, thereby enhancing DNA binding affinity and promoting transcriptional activation of zein biosynthesis. These findings unveil a novel regulatory mechanism underlying the coordinated synthesis of starch and zeins in maize endosperm.

Arabidopsis SnRK2.6 (OST1) regulates hormonal and metabolic control of leaf starch turnover, but its role in storage‐starch synthesis remains unclear (Zheng et al. 2010). In cassava, ABA‐induced MeSnRK2.3 phosphorylates MebHLH68 to regulate starch synthesis genes (Li et al. 2024). In this study, our integrated multi‐omics and biochemical analyses indicated that subclass III SnRK2s primarily regulate starch synthesis through posttranslational phosphorylation of starch synthesis‐related enzymes (SSREs) (Figure 2i,j, Figures S11c–e and S12). In cereal endosperm, although phosphorylation of SSREs has been extensively documented (Grimaud et al. 2008; Mehrpouyan et al. 2021; Pang et al. 2018; Tetlow et al. 2004; Walley et al. 2013), direct genetic evidence linking this modification to final starch accumulation remains lacking. Here, we show that ZmSnRK2.10 directly phosphorylates Bt1 at Ser141, enhancing its ADPG transport activity and significantly increasing the final starch content in mature kernels (Figure 3). Bt1 is a plastid membrane‐localized protein spanning the amyloplast envelope (Kirchberger et al. 2007), whereas ZmSnRK2.10 resides primarily in the endosperm cytoplasm and is not a membrane protein (Figures S15 and S25). Notably, subclass III SnRK2 kinases regulate plastid membrane‐localized substrates at the cytosol–plastid membrane interface, including SLAC1 (anion channel; Lee et al. 2009), KAT1 (K^+^ channel; Sato et al. 2009), RbohF (NADPH oxidase; Sirichandra et al. 2009), EGR2 (clade‐E growth‐regulating 2; Ding et al. 2019), and OsCNGC9 (Ca^2+^ channel; Wang et al. 2021). Consistent with this paradigm, Bt1 contains a cytoplasmic‐facing SnRK2 recognition motif (R‐X‐X‐S) encompassing Ser141, the identified phosphorylation site (Figure S17). Furthermore, ZmSnRK2.10 and ZmSnRK2.8 appear to interact with Bt1 around the plastid, suggesting that phosphorylation occurs on the cytoplasmic face of the plastid envelope (Figure 3b, Figures S15 and S16a). Moreover, the Ser141 site is highly conserved among Bt1 homologs (Figure S17b), implying that this phosphorylation‐dependent regulatory mechanism may be widespread across crops. These evolutionarily conserved phosphorylation sites are particularly intriguing because they may regulate starch biosynthetic enzymes, thereby controlling starch content and yield for specific applications.

Additionally, in cereal endosperm, starch synthesis involves core SSREs functioning through their association in heteromeric complexes. The cytosolic AGPase is a heterotetramer composed of two large subunits (Sh2 protein) and two small subunits (Bt2 protein) (Hannah and Boehlein 2017). Furthermore, several of the core SSREs, like SSs, SBEs, and DBEs, assemble into coordinated heteromeric complexes within the amyloplast, where SSIII physically associates with other enzymes, including PPDKs, and the assembly of these complexes is regulated by protein phosphorylation (Hennen‐Bierwagen et al. 2009; Mehrpouyan et al. 2021). Quantitative phosphoproteomics revealed multiple phosphorylation sites on core SSREs and identified Bt2, SSIII, GBSSI, SBEI, Zpu1, and PPDKs as substrates whose phosphorylation depends on subclass III SnRK2 kinases (Figure 2j and Figure S13). Moreover, IP–MS and AI–PPI analyses indicate that these SSREs and PPDKs interact with ZmSnRK2.10, either directly or indirectly (Figure S14). Using BiFC and LCI assays, we confirmed the direct interaction between ZmSnRK2.10 and both GBSSI and PPDK2, but not with Bt2 or SBEI (Figure S19b,c). These findings suggest that some of these core SSREs are potential substrates of subclass III SnRK2s. However, due to the large size of these proteins, further biochemical assays and comparative studies are necessary to elucidate the specific phosphorylation mechanisms. Notably, immunofluorescence assays demonstrate that certain proteins reported to be in amyloplast‐localized complexes normally reside in the cytosol (Figure S19d). These results indicate that ZmSnRK2.10 may phosphorylate these enzymes in the cytoplasm before their transport to the amyloplast for starch synthesis. A similar mechanism is reported in Arabidopsis, where the chloroplast‐localized PPD5 is phosphorylated by SnRK2.6/OST1 in the cytoplasm before its transport to the chloroplast (Hong et al. 2020). Collectively, these findings suggest that the phosphorylation of these core SSREs by subclass III SnRK2s may enhance both their catalytic activity and complex formation, thereby promoting starch synthesis in maize endosperm.

Synthesis of zeins exhibits spatiotemporally specific expression patterns and is primarily regulated at the transcription level (Yang et al. 2023). Our integrated omics analysis demonstrates that subclass III SnRK2s regulate the synthesis of zeins at the transcriptional level (Figure 2h). O2, a master TF for endosperm zein synthesis (Schmidt et al. 1987), exhibits significantly enhanced phosphorylation in *zmsnrk2.8;2.9;2.10* (Figure 4a,b). O2 phosphorylation at Ser210 and Ser212 markedly impairs its binding to the 22‐kD *α‐zein* promoter (Figure 4c–e), consistent with previous studies showing that O2 hyperphosphorylation inhibits its DNA binding activity (Ciceri et al. 1997). The hyperphosphorylation of protein O2 in the mutant kernels likely results from subclass III SnRK2‐mediated inhibition of other kinases (e.g., GSK11, CDK2, and CDK10) targeting Ser210/Ser212, as supported by NetPhos3 predictions and multi‐omics data (Figure S21, Table S7). This contrasts with SnRK2.2, which directly phosphorylates O2 at Thr387 to enhance its transactivation of zein genes under ABA signaling (Yang, Wang, Guo, et al. 2022). Furthermore, a recent study demonstrated that SnRK1α1 directly phosphorylates O2 at Ser21, promoting its degradation and suppressing zein synthesis under low sucrose conditions (Yang et al. 2024). Conversely, sucrose‐activated ZmSnRK2.10 indirectly maintains O2 hypophosphorylation at Ser210/Ser212, thereby enhancing its DNA binding to zein promoters. These distinct phosphorylation sites indicate that SnRK1 and SnRK2s regulate O2 through parallel pathways. Additionally, sucrose‐activated ZmSnRK2.10 may also suppress SnRK1‐mediated phosphorylation of O2 at Ser21 (Yang et al. 2024) indirectly via recruitment of PP2C, as reported in Arabidopsis (Belda‐Palazón et al. 2020). Collectively, these findings highlight a sophisticated kinase network through which SnRK1 and SnRK2s fine‐tune zein biosynthesis in response to ABA, sucrose, and energy status. Moreover, they suggest potential crosstalk between SnRK2s and the SnRK1 pathway in maintaining nutritional homeostasis in maize endosperm, although the underlying mechanisms require further exploration.

### 3.3 ZmSnRK2.10 Mediated Sucrose and ABA to Regulate Endosperm Filling and Embryo Development, Respectively

ABA levels in developing maize endosperm (or kernels) exhibit a bimodal distribution characterized by two distinct peaks. The first peak occurs immediately prior to kernel filling initiation (Jones and Brenner 1987; Wang et al. 2025). Yang, Wang, Guo, et al. (2022) demonstrated that ABA‐induced SnRK2.2 phosphorylates transcription factors bZIP29, ABI19, and O2, enhancing their transactivation potential to initiate endosperm filling. In contrast, we reveal that ZmSnRK2.10 plays a distinct role through sequential activation: it is activated by sucrose in the endosperm and by ABA in the embryo during kernel development. This establishes sucrose‐dependent activation of SnRK2.10 as a mechanistically distinct pathway relative to ABA‐induced SnRK2.2 activation during maize endosperm development. Supporting this model, ZmSnRK2.10 undergoes significant phosphorylation during endosperm filling (Figure 5b), coinciding with minimal endospermic ABA levels at this stage (Jones and Brenner 1987). This temporal pattern suggests that ABA is unlikely to activate ZmSnRK2.10 during filling. Instead, high sucrose levels during early kernel filling (Figure 5d) correlate with ZmSnRK2.10 phosphorylation and autophosphorylation activity (Figure 5b). Exogenous sucrose application significantly enhances ZmSnRK2.10 phosphorylation and kinase activity (Figure 5e and Figure S26). Notably, analyses of maize mutants defective in sucrose accumulation (*zmsugcar1*) and ABA accumulation (*dg1*) demonstrate that sucrose enhances ZmSnRK2.10 phosphorylation independently of ABA (Figure 5f,g). While sucrose acts as both an essential metabolic substrate and signaling molecule in endosperm development, the underlying signaling mechanisms remain poorly characterized. Although elevated sucrose was recently shown to stimulate endosperm filling through SnRK1α1 suppression (Yang et al. 2024), our study reveals a novel pathway in which sucrose activates ZmSnRK2.10 to enhance maize endosperm filling. Although subclass III SnRK2s can be activated by osmotic stressors like sucrose in Arabidopsis (Boudsocq et al. 2004), our study establishes sucrose as a developmental signal activating ZmSnRK2.10 specifically in endosperm to modulate storage synthesis. This sucrose–ZmSnRK2.10 module functions not as a stress response, but as a fundamental physiological mechanism controlling grain yield and quality.

ABA‐mediated SnRK2s are essential for storage reserve accumulation and the acquisition of desiccation tolerance during embryo development in Arabidopsis (Nakashima et al. 2009; Nonogaki 2019). In maize embryos, ABA levels increase progressively during late developmental stages (Figure 5d), temporally coinciding with enhanced phosphorylation of ZmSnRK2.10 and increased autophosphorylation activity (Figure 5c). Consistent with previous findings (Long et al. 2021), exogenous ABA significantly enhances ZmSnRK2.10 phosphorylation and kinase activity (Figure 5e and Figure S26). In maize, transcription of globulin‐encoding genes (*Glbs*) is primarily activated by ABA. This activation in the embryo is induced by suppression of zein biosynthesis in the endosperm, reflecting endosperm–embryo proteomic rebalancing (Rivin and Grudt 1991; Zheng et al. 2019). However, our findings demonstrate that *zmsnrk2.8;2.9;2.10* mutants exhibit significant reductions in both zeins and GLBs (Figure 1h–k), with subclass III SnRK2 co‐regulating the biosynthesis of these storage proteins in maize at the transcriptional level (Figure 2h and Figure S11a,b). Furthermore, mutations in all four subclass III SnRK2s result in severe vivipary (Figure 1a), a phenotype analogous to that observed in the Arabidopsis *snrk2.2/2.3/2.6* mutant (Nakashima et al. 2009). The GLBs deficiency and viviparous defects likely stem from a complete blockade of ABA signaling in mutant embryos. Subclass III SnRK2s function by phosphorylating downstream A‐group bZIP transcription factors (TFs) to regulate ABA responses in seeds (Johnson et al. 2002; Kobayashi et al. 2005; Nakashima et al. 2009). Supporting this mechanism, we observed significantly decreased phosphorylation levels of several A‐group bZIP TFs, including ZmbZIP75 (a homolog of ABI5), in the *zmsnrk2.8;2.9;2.10* kernels (Figure 4a). Consistent with our previous findings, we establish that ZmSnRK2.10 directly phosphorylates ZmbZIP75 at Ser‐129 to enhance its transcriptional activity (Long et al. 2024). Transient co‐expression assays further confirmed the synergistic activation of *Glb1* transcription by ZmSnRK2.10 and ZmbZIP75 (Figure 4f). The vivipary phenotype in SnRK2 mutants, coupled with ABA‐responsive phosphorylation of A‐group bZIP transcription factors, demonstrates that subclass III SnRK2 kinases mediate ABA signaling not only for storage protein synthesis but also for late embryogenesis and dehydration tolerance. This role is consistent with their conserved function in seed maturation (Nakashima et al. 2009; Wang et al. 2025). Collectively, these findings confirm that the core “ABA‐SnRK2s‐bZIPs” pathway, governing storage protein synthesis and late embryogenesis programs, is evolutionarily conserved across both monocots and dicots.

**FIGURE 7:**
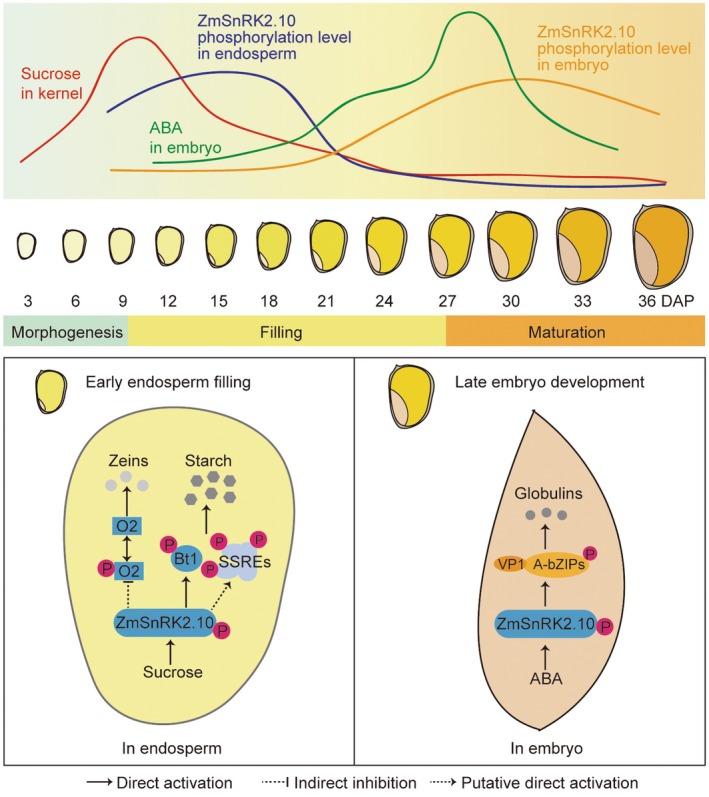
A proposed model for the sequential activation of ZmSnRK2.10 by sucrose and ABA in regulating kernel filling in maize. During the early filling stage, maize kernels exhibit low abscisic acid (ABA) levels and high sucrose concentrations. As the kernels enter this stage, ZmSnRK2.10 is activated by elevated sucrose levels in the endosperm. This sucrose‐activated ZmSnRK2.10 interacts with and phosphorylates the ADP‐glucose transporter Bt1, along with several starch synthesis‐related enzymes, thereby enhancing their activity and promoting starch accumulation. Additionally, ZmSnRK2.10 indirectly downregulates the phosphorylation of O2 transcription factor (TF), enhancing its transactivation of downstream zein genes. In the later stages of kernel development, ZmSnRK2.10 is activated by elevated ABA concentrations in the embryo, and then phosphorylates A‐group bZIP TFs to regulate globulin synthesis and late embryo development in cooperation with VP1. In summary, ZmSnRK2.10 functions as a key regulator, orchestrating sucrose and ABA signaling to control both endosperm filling and late embryo development in maize kernels.

In summary, our work demonstrates that subclass III SnRK2s function redundantly in modulating kernel filling through coordinated regulation of starch and storage protein synthesis, with ZmSnRK2.10 playing a primary role. Significantly, we unveil a spatiotemporally ordered activation mechanism underlying subclass III SnRK2‐mediated control of kernel filling in maize. In the endosperm, ZmSnRK2.10 is activated by high sucrose during early kernel development, which then promotes starch and zein synthesis through two distinct pathways. Sucrose‐activated ZmSnRK2.10 directly phosphorylates SSREs, such as Bt1, to facilitate starch synthesis, while concomitantly suppressing O2 phosphorylation through indirect mechanisms, thereby enhancing its DNA binding ability to promote zein synthesis. In the embryo, ZmSnRK2.10 is activated by elevated ABA levels during late kernel development, which subsequently regulates GLB synthesis and embryo development via the conserved “ABA‐SnRK2s‐bZIPs” pathway (Figure 7). Additionally, overexpression of *ZmSnRK2.10* in both inbred and hybrid backgrounds significantly increases grain yield, accompanied by enhanced starch and protein accumulation, suggesting its potential for yield and quality improvement.

## 4 Materials and Methods

### 4.1 Plant Materials and Growth Conditions

All maize materials were cultivated on the farm at Sichuan Agricultural University in Sichuan, China, and in the field in Sanya, China. For CRISPR‐Cas9 knockout mutants, the specific gRNAs of *ZmSnRK2.8/2.9/2.10/2.12* were designed by CRISPR‐P version 2.0 (http://crispr.hzau.edu.cn/cgi‐bin/CRISPR2/CRISPR), according to the high similarity in gene sequences. The CRISPR‐Cas9 knockout lines of *ZmSnRK2.8/2.9/2.10/2.12* were obtained separately in the KN5585 background through *Agrobacterium tumefaciens* ‐mediated transformation. The knockout plants were backcrossed with KN5585 twice and then self‐pollinated to obtain heterozygous lines without the Cas9 cassette by polymerase chain reaction (PCR) amplification. The single knockout mutants *zmsnrk2.8*, *zmsnrk2.9*, *zmsnrk2.10*, and *zmsnrk2.12* were hybridized in different combinations to create double, triple, and quadruple knockout mutants, resulting in a total of fifteen distinct mutant types. For the overexpression transgenic lines, the full‐length coding sequences (CDS) of *ZmSnRK2.10*, *Bt1*, and phospho‐mimetic *Bt1* ^*S141D*^ were tagged with 3 × Flag at the 3′ terminus, cloned into the pCAMBIA3301 vector, and driven by the *Ubiquitin* promoter, respectively. The *ZmSnRK2.10* overexpression lines were generated in the B104 background through *Agrobacterium tumefaciens* ‐mediated transformation. The *Bt1* and *Bt1* ^*S141D*^ overexpression lines were generated in the KN5585 background through *Agrobacterium tumefaciens* ‐mediated transformation. The ethyl methanesulfonate (EMS)‐induced mutants of *dg1* (EMS4‐006287) and *zmsugcar1* (EMS4‐144e9f) were obtained from the public EMS mutant library (http://maizeems.qlnu.edu.cn/).

The developing kernels were harvested at 3, 6, 9, 12, 15, 18, 21, 24, 27, 30, 33, and 36 days after pollination (DAP) for real‐time PCR, western blot, and abscisic acid (ABA), sucrose, and starch content measurement. The 15‐DAP kernels were collected and treated with varying concentrations of sucrose and ABA (0, 100, 200, and 400 mM sucrose and 100 μM ABA) according to our previous research (Li et al. 2018). All these samples were collected from three individual ears for each developmental stage and immediately frozen in liquid nitrogen and stored at −80°C.

### 4.2 Genotyping

To identify the transgenic materials, leaves were harvested, and the genomic DNA was extracted using the cetyl trimethylammonium bromide (CTAB) method. For CRISPR‐Cas9 knockout mutants, the Cas9 cassette was detected by PCR identification with specific primers listed in Table S8. The Cas9‐target sites were amplified and analyzed by sequencing. For the overexpression materials, the sequences of *ZmSnRK2.10* were amplified from genomic DNA using specific primers to genotype the transgenic materials. The overexpression level was analyzed by quantitative real‐time PCR (qRT‐PCR); the primers are listed in Table S8. The flag‐fused protein was detected by western blot using anti‐Flag antibody (Sigma, F1804, 1:5000 dilution).

### 4.3 Physiological Analyses of Maize Kernels

Mutant, overexpression (OE), and wild‐type (WT) plants were grown together in the same field plot. Phenotypically uniform plants of each genotype were tagged and pollinated synchronously. Well‐pollinated ears from nonborder rows were harvested, and kernels from the central region of the ears were selected for 10‐ or 100‐kernel weight measurements. Only kernels with consistent embryo orientation, intact pericarps, and uniform coloration were included. Mature kernels were ground into fine powders to measure starch, soluble sugar, and protein content. Starch content was determined according to the manufacturer's instructions (Megazyme Total Starch Kit; KTSTA‐50A). Soluble sugar was determined according to the soluble sugar kit instructions (Solarbio; bc0030). To extract zein proteins, a total of 50 mg fine powder from each sample was prepared and incubated in 0.5 mL zein extraction buffer (3.75 mM sodium borate, 2% 2‐mercaptoethanol [v/v], 0.3% SDS [w/v], and 70% ethanol [v/v]; pH 10). The non‐zein protein was extracted by non‐zein extraction buffer (2.5 mM sodium borate, 2% 2‐mercaptoethanol [v/v], and 5% SDS [w/v]; pH 10). The zein and non‐zein accumulation were visualized by 15% SDS‐PAGE gel electrophoresis and quantified by using a BCA protein assay kit (Coolaber, SK1070). The 15‐DAP immature seeds were collected for metabolic measurements using the LC‐MS/MS system analysis according to a previously described method (Yang et al. 2018). The starch in maize kernels was examined using I_2_/KI staining, as described previously (Li et al. 2018). Sucrose content was determined in different DAP of B73 kernels, according to the manual of the kit (Geruisi‐bio, G0545F) by the hexokinase method.

### 4.4 Cytological Analysis

For light microscopy analysis, the mature grains were cut transversely, and the peripheral and central regions of the endosperm were observed using a stereomicroscope (Olympus; SZX10). The immature kernels at 15‐DAP were fixed in FAA buffer (formaldehyde: acetic acid: ethanol: water = 10:5:50:35 [v:v:v:v]) for paraffin sectioning. The sections were stained with 0.1% magenta solution and then photographed using a Leica DM IL LED microscope.

For scanning electron microscope (SEM) analysis, the mature grains were cut longitudinally in the middle of the embryos, and starch granules of starchy endosperm were observed using a scanning electron microscope (Quanta450). For transmission electron microscope (TEM) analysis, the immature kernels at 15‐DAP were sliced and fixed in phosphate buffer (pH 7.2) with 2.5% (w/v) glutaraldehyde. The sections were examined and photographed using a transmission electron microscope (Hitachi; H7600).

### 4.5 Expression Pattern and Protein Localization

The developing kernels were collected at 3, 6, 9, 12, 15, 18, 21, 24, 27, 30, 33, and 36 DAP from maize inbred line B73 for qRT‐PCR. RNA extraction was performed according to the protocol of the FastPure Universal Plant Total RNA Isolation Kit (Vazyme; RC411‐01). Total RNA (1 μg) was used for first‐strand cDNA synthesis according to the HiScript II Q RT SuperMix kit (Vazyme; R212‐02). qRT‐PCR experiments were performed on a CFX96 Real‐Time System (Bio‐Rad, USA) according to the instructions of ChamQ Universal SYBR qPCR Master Mix (Vazyme; Q711‐02). Three independent biological replicates from three different ears were performed. The relative gene expression was analyzed according to the $2^{–\Delta \Delta C_{t}}$ method, and maize *β‐actin* was used as the internal reference gene. All primer details are listed in Table S8.

The protein extraction and immunoblot analyses were performed as described previously (Long et al. 2021). For protein accumulation analysis, seeds (3–36 DAP), endosperms (9–36 DAP), and embryos (12–36 DAP) were collected at 3‐day intervals from the maize inbred line B73. Total protein of these tissues was extracted by lysis buffer (50 mM Tris–HCl, pH 7.6; 150 mM NaCl; 5 mM MgCl_2_; 0.5% NP‐40; 0.5 mM DTT; 1 mM PMSF; 1× proteinase inhibitor cocktail). Total protein (20 μg) was separated by 10% SDS‐PAGE. The expression of ZmSnRK2.8/2.10 at the protein level was detected by western blot using ZmSnRK2.8/2.10‐specific antibodies.

For protein localization, immunofluorescence was performed as described (Qin et al. 2021) with technical assistance from Servicebio (Wuhan, China). Longitudinal sections (~1 mm thick) were prepared from 15‐ and 25‐DAP kernels of the B73 inbred line and incubated with or without anti‐ZmSnRK2.10 antibody. Similarly, 15‐DAP kernels from KN5585 (wild‐type) and the *zmsnrk2.8;9;10* triple mutant were sectioned in the same manner and incubated with anti‐ZmSnRK2.10 antibody in combination with antibodies against SSREs. All samples were observed under a confocal laser scanning microscope (LSM880, Carl Zeiss) with an excitation wavelength of 488 nm.

### 4.6 Polyclonal Antibody Generation, Immunoblot, and Phos‐Tag Mobility Shift Assay

To generate anti‐ZmSnRK2.8 and anti‐ZmSnRK2.10 antibodies, the full‐length CDS of *ZmSnRK2.8* and *ZmSnRK2.10* were amplified and cloned into the pGEX‐6t vector. Both antibodies were produced in rabbits by ABclonal (Wuhan, China, 1:5000 dilution).

Total protein from WT and *zmsnrk2.8/2.9/2.10* seed at 15‐DAP was extracted in the same way and was detected by western blot using different specific antibodies. The anti‐Glb (1:2500 dilution), anti‐Sh1 (1:4000 dilution), and anti‐GBSSI (1:2500 dilution) antibodies were presented by Wu Lab (Zhang et al. 2016; Zheng et al. 2019). The anti‐O2 (1:5000 dilution), anti‐Bt1 (1:5000 dilution), and anti‐SSIIa (1:2500 dilution) antibodies were presented by Zhang Lab (Chen et al. 2023). The anti‐Bt2 (1:2500 dilution), anti‐Sh2 (1:2500 dilution), anti‐SBEIIb (1:2500 dilution), anti‐Zpu1 (1:2500 dilution), and anti‐PPDK (1:1250 dilution) antibodies were purchased from Orizymes (Shanghai, China). The anti‐phospho‐S175‐SnRK2 (AP1481, 1:500 dilution), anti‐actin (AC009, 1:10000 dilution), HRP Goat Anti‐Rabbit IgG (H + L) (AS014, 1:5000 dilution), and HRP Goat Anti‐Mouse IgG (H + L, 1:5000 dilution) (AS003) antibodies were purchased from ABclonal (Wuhan, China). The phosphorylated protein bands were detected using 10% (w/v) SDS–PAGE gel containing 50 μM phos‐tag reagent (Wako; AAL‐107) and 100 μM MnCl_2_. The protein bands were quantified using ImageJ software according to the manufacturer's instructions.

### 4.7 Transcriptome Profiling

The total RNA was extracted from 15‐DAP kernels of homozygous *zmsnrk2.8;9;10* and WT, which were harvested from three individual ears, separately. The RNA libraries were constructed and sequenced by APTBIO (Shanghai, China). The RNA library quality was assessed on an Agilent Bioanalyzer 4150 system, and the library preparations were sequenced on an Illumina Novaseq 6000 (or MGISEQ‐T7). The data generated from the Illumina (or BGI) platform were used for bioinformatics analysis. The differentially expressed genes (DEGs) were identified with the thresholds of a |log_2_FC (fold change)| > 1.0 and *p*‐value < 0.01. GO function enrichment was analyzed by the program Phyper (http://www.geneontology.org/), and KEGG pathway enrichment was also performed using KEGG Mapper (https://www.kegg.jp/kegg/tool/map_pathway2.html).

### 4.8 Quantitative Proteomics and Phosphoproteomics

#### 4.8.1 Protein Extraction and Digestion

The total protein was extracted from 15‐DAP kernels of homozygous *zmsnrk2.8;9;10* and WT, each from three individual ears. Protein was quantified using the BCA protein assay kit (Coolaber, SK1070). Protein digestion by trypsin was performed according to the filter‐aided sample preparation (FASP) procedure. The SDS, DTT, and other small molecules were removed with UA buffer (8 M Urea, 150 mM Tris–HCl pH 8.0). Iodoacetamide (100 mM IAA in UA buffer) was added to block reduced cysteine residues, and samples were incubated for 30 min in the dark. The filters were washed with UA buffer and 25 mM NH_4_HCO_3_ buffer. After digestion, peptides were collected, desalted, concentrated, and reconstituted in 0.1% (v/v) formic acid. The peptide concentration was estimated by UV light spectral density at 280 nm, using an extinction coefficient of 1.1 of 0.1% (g/l) solution.

#### 4.8.2 Labeling and Fractionation

The peptide mixture of each sample was labeled using TMT (Thermo Scientific) reagent following the manufacturer's instructions. The labeled peptides were fractionated by Strong Cation Exchange (SCX) chromatography using the AKTA Purifier system (GE Healthcare). The dried peptide mixture was reconstituted, acidified with buffer A (10 mM KH_2_PO_4_ in 25% ACN, pH 3.0), and loaded onto a PolySULFOETHYL column (PolyLC Inc.). The peptides were eluted with a gradient of buffer B (500 mM KCl, 10 mM KH_2_PO_4_ in 25% ACN, pH 3.0) at a flow rate of 1 mL/min. The elution was monitored by absorbance at 214 nm, with fractions collected every minute. The collected fractions were desalted and concentrated for MS analysis.

#### 4.8.3 Phosphopeptides Enrichment

The TMT‐labeled phosphopeptides were enriched using Titansphere Phos‐TiO_2_ beads (GL Sciences 5010–21315). The samples were reconstituted in precooled IAP Buffer, mixed with TiO_2_ beads, agitated for 40 min, and centrifuged. The supernatant was discarded, and then the beads were washed with washing buffer. The phosphopeptides were eluted with elution buffer, concentrated under vacuum, and dissolved in 0.1% formic acid for MS analysis.

#### 4.8.4 Liquid Chromatography Tandem Mass Spectrometry (LC–MS/MS) Analysis

LC–MS/MS analysis was performed using a Q Exactive HF mass spectrometer (Thermo Scientific) that was coupled to Easy nLC (Proxeon Biosystems, now Thermo Fisher Scientific) for 120 min. The peptides were loaded onto a reverse‐phase trap column (Thermo Scientific) connected to the C18‐reversed phase analytical column (Thermo Scientific) in buffer A (0.1% formic acid) and separated with a linear gradient of buffer B (84% acetonitrile and 0.1% formic acid) at a flow rate of 300 nL/min. The mass spectrometer was operated in positive ion mode. MS data were acquired using a data‐dependent top10 method dynamically choosing the most abundant precursor ions (300–1800 m/z) for HCD fragmentation. Key settings included a 70 000 resolution for MS, 17 500 for HCD, AGC target of 3e6, 10 ms maximum injection time, 2 m/z isolation width, 30 eV normalized collision energy, 0.1% underfill ratio, and a 40‐s dynamic exclusion. Peptide recognition mode was enabled.

### 4.9 Protein–Protein Interaction Assays

The IP–MS method and LC–MS/MS analysis of immunoprecipitated proteins were performed according to a method described previously (Lang et al. 2015). The extracted protein from 15–DAP B73 kernels was incubated with anti‐ZmSnRK2.10 antibody and Pierce protein A Magnetic Beads (Thermo Scientific; #88802) at 4°C for 4 h with gentle agitation. IgG was used as the negative control. Next, the beads were washed with PBS containing 1% Nonidet P‐40, finally diluted in lysis solution (4% SDS; 100 mM Tris; 1 mM DTT) and boiled for 10 min. The samples were analyzed through mass spectrometry detection by Oebiotech (Shanghai, China).

For the bimolecular fluorescence complementation (BiFC) assay, the coding regions of *ZmSnRK2.8/2.10* were fused to a pRTVcVN vector, while the *SSRGs and O2* were cloned into a pRTVcVC vector. Equal amounts of the combined plasmids (5 μg each) were transformed into maize leaf protoplasts. The YFP signal was observed using a scanning confocal microscope (Leica, STELLARIS STED/EM CPD300) after 24 h dark incubation.

For the luciferase complementation imaging (LCI) assay, the coding regions of *ZmSnRK2.8/2.10* were cloned into a pCAMBIA1300‐nLuc vector, while the *SSRGs and O2 were* cloned into a pCAMBIA1300‐cLuc vector. These constructs were transformed into Agrobacterium strain GV3101, and different combinations were subsequently transfected into the *N. benthamiana* leaves. *Nicotiana benthamiana* plants were grown in a greenhouse at a constant temperature of 22°C and a humidity of 60% under a 16‐h light and 8‐h dark photoperiod. The Luc images of infiltrated leaves were obtained after 2 days of cultivation by the ChemiDoc System (Bio‐Rad).

For the GST pull‐down assay, the CDSs of *ZmSnRK2.10* and *ZmSnRK2.8* were cloned into plasmid pGEX‐6T. Recombinant proteins GST‐ZmSnRK2.10, GST‐ZmSnRK2.8, and GST were expressed in *E. coli* Rosetta (DE3) cells and purified using GST‐tag Purification Resin (Beyotime, P2251). Separately, the CDS of *Bt1* was cloned into plasmid pET32a, and recombinant protein His‐Bt1 was expressed in *E. coli* Rosetta (DE3) cells and purified using His‐tag Purification Resin (Beyotime, P2233). For the pull‐down assay, 1 μg of purified GST‐ZmSnRK2.10, GST‐ZmSnRK2.8, or GST was individually incubated with Glutathione Sepharose beads at 4°C for 2 h. A total of 1 μg of purified His‐Bt1 protein was then added and incubated for an additional 2 h. The beads were washed five times with 1× PBS buffer. Bound proteins were eluted in SDS loading buffer, separated by SDS‐PAGE (10% acrylamide gel), and detected by immunoblotting using anti‐GST (ABclonal, AE077) and anti‐His (ABclonal, AE086) antibodies.

### 4.10 Transient Expression Assays by Particle Bombardment

The assay was achieved following the protocol of our previous report (Li et al. 2018). Both reporter and effector vectors were constructed, based on the modified plant expression vector pBI221. The reporter vectors were created by integrating the promoters (⁓1500 bp upstream of the ATG) of 22‐KD *α‐zein* and *Globulin1* into the upstream region of Luc, while the effector vector was constructed by placing ZmSnRK2.10, ZmbZIP75, VP1 and O2, as well as the site‐directed mutants O2^S210AS212A^ and O2^S210DS212D^, under the control of the *ubiquitin* promoter. The Gus reference vector was driven by the *ubiquitin* promoter and used as an internal control. The bombarded endosperms were hatched in darkness for 24 h at 28°C to determine GUS and LUC activities.

### 4.11 Measurements of SSRE Activities and Bt1 Transport Activity

The immature kernels at 15‐DAP of *zmsnrk2.8;9;10*, *ZmSnRK2.10*‐OE, and their corresponding WT were harvested and used for crude protein extraction, followed by the determination of the activities of AGPase, SS, SBE, and DBE using the corresponding assay kits (Suzhou Comin Biotechnology Co. Ltd., Suzhou, China).

Since Bt1 transports one molecule of ADPG into the cell while simultaneously excluding one molecule of ADP from the amyloplast, the transport activity of the Bt1 protein was determined by measuring the ADP content in the solution. Amyloplasts were isolated from developing maize endosperm according to the previously published methods (Echeverria et al. 1985), and the ADPG transport assay was performed as previously described (Cakir et al. 2016; Kirchberger et al. 2007). Briefly, the amyloplast isolation was performed from 15‐DAP endosperm of WT, *zmsnrk2.8, zmsnrk2.10*, and *zmsnrk2.8;9;10*, respectively. Then the isolated amyloplasts (200 μL) were transferred into a reaction mixture buffer (100 mM Bicine, 0.5 M sorbitol, 12.5 mM EDTA, 50 mM potassium acetate, 10 mM glutathione, 5 mM ADPG, and 20 mM ATP). The ADP content was measured after 6‐min incubation (three biological replicates). ADP content was determined using the ADP–Glo Kinase Assay (Promega) according to the manufacturer's instructions.

In addition, equal amounts of the purified His‐Bt1, His‐Bt1^S141D^, and His‐Bt1 with GST‐ZmSnRK2.10 proteins were added into the amyloplast isolated from WT endosperm at 15‐DAP. Then, they were transferred into a reaction mixture, and the ADP content was measured after 0, 2, 4, and 6 min of incubation (three biological replicates for each time point).

### 4.12 Transcriptional Activation Analysis

The sequences of O2, O2^S210AS212A^, and O2^S210DS212D^ were amplified and cloned into the vector pGBKT7. The empty vector pGBKT7 was used as the negative control. All constructs were transformed into yeast strain AH109 using the PEG/LiAc method. Transformants were selected on SD/−Trp medium and subsequently screened on SD/−Trp–His–Ade medium, following the Yeast Protocols Handbook. For the dual‐luciferase reporter assay, the same sequences were inserted into the GAL4‐DB vector driven by the CaMV *35S* promoter as effectors. The *Luc* gene, driven by the *35S* mini promoter containing a 5×Gal4 binding site, was used as the reporter. The *Renilla Luc* gene, driven by the *35S* promoter, served as the reference. The empty vector was used as the negative control. These plasmids were transfected into maize leaf protoplasts, and the ratio of Luc/Ren activity was determined as described previously (Li et al. 2018).

### 4.13 Electrophoretic Mobility Shift Assay (EMSA)

Oligonucleotide probes were synthesized and labeled by Sangon Biotech (Shanghai, China). The His‐fused O2, O2^S210AS212A^, and O2^S210DS212D^ were expressed in *E. coli* Rosetta (DE3) cells and purified using His‐tag Purification Resin (Beyotime, P2233). The EMSA assays were carried out following the protocol of the LightShift Chemiluminescent EMSA kit (Thermo Scientific). Briefly, the recombinant protein was incubated with biotin‐labeled probe at 25°C for 30 min, and the reaction results were separated on a native‐PAGE gel. The binding signal was visualized using SuperSignal West Femto (Thermo Scientific, 34095).

### 4.14 In‐Gel Kinase Assay

The In‐gel kinase assay was performed as described previously (Ding et al. 2015). Total proteins were extracted from 15‐DAP kernels after sucrose and ABA treatment in gel protein extraction buffer (25 mM HEPES–KOH pH 7.5, 5 mM EDTA pH 8.0, 5 mM EGTA pH 8.0, 25 mM NaF, 1 mM Na_3_VO_4_, 20% (v/v) glycerol, 10 mM DTT, 1 mM PMSF, and 1× proteinase inhibitor cocktail). Proteins were immunoprecipitated with Protein G agarose beads (Invitrogen), which were conjugated with ZmSnRK2.10 antibody, then separated on an SDS‐PAGE (10% acrylamide) gel containing MBP substrate. The phosphorylated bands were detected by autoradiography (Typhoon 9410 imager).

### 4.15 In Vitro Phosphorylation Assay

The in vitro phosphorylation assay was performed as described previously (Yang, Wang, Guo, et al. 2022). A total of 10 μg recombinant protein His‐Bt1 or His‐Bt1^S141A^ was incubated with 1 μg GST‐ZmSnRK2.10 or GST‐ZmSnRK2.8 in the reaction buffer (50 mM Tris–HCl at pH 7.5, 10 mM MgCl_2_,1 mM DTT, and 1 mM ATP) at 30°C for 2 h; then the reactions were terminated and separated on a 10% (v/w) SDS‐PAGE gel with 50 mM of Phos‐tag (Wako; AAL‐107) and 100 μM MnCl_2_. Immunoblot analysis was performed according to the protocol provided by Wako Chemicals and detected with anti‐His antibody (ABclonal, AE086).

### 4.16 Statistics and Analysis

GraphPad Prism 8 was used for statistical analysis of one‐way ANOVA and Tukey's test. SPSS version 26.0 was used for Duncan multiple range test. Microsoft Excel 2019 was used for two‐tailed Student's *t*‐test analysis.

## Author Contributions

Conceptualization, Y.L. (Yangping Li), and Y.H. (Yubi Huang); Methodology, T.L., Y.W., C.M., S.F., Q.L., and H.L.; Investigation, Y.L. (Yinghong Liu), Y.H. (Yufeng Hu), L.G., C.C., and X.F.; Writing‐Original Draft, Y.L. (Yangping Li), and T.L.; Writing‐Review and Editing, B.X., J.Z., and D.Z.; Project administration, Y.L. (Yangping Li), and Y.H. (Yubi Huang).

## Funding

The work was supported by grants from the National Key R&D Program of China (2021YFF1000304), the National Natural Science Foundation of China (32201696), the Natural Science Foundation of Sichuan Province (2022NSFSC0016), and the Sichuan Maize Innovation Team Program (SCCXTD‐2024‐02).

## Conflicts of Interest

The authors declare no conflicts of interest.

## Supporting information


**Figure S1:** CRISPR‐Cas9‐edited sequence of the ZmSnRK2.8/9/10/12 mutants.
**Figure S2:** Phenotypic analysis of the WT and zmsnrk2s mutants.
**Figure S3:** Kernel weights and major storage compound contents of WT and zmsnrk2 mutants.
**Figure S4**. THE qRT‐PCR analysis of the expression profiles of subclass III *ZmSnRK2* genes in developing maize kernels.
**Figure S5:** Measurement of dry weight during developing kernels of WT and zmsnrk2.8;9;10.
**Figure S6:** Paraffin sections of 10‐, 12‐, and 15‐DAP kernels of WT and zmsnrk2.8;9;10.
**Figure S7:** Metabolome analysis of 15‐DAP WT and zmsnrk2.8;9;10 kernels.
**Figure S8:** Characteristics of the omics data analyses.
**Figure S9:** Characteristics of the comparative phosphoproteome in WT and zmsnrk2.8;9;10 filling kernels.
**Figure 10**. Comparison and relationship of transcriptome, proteome, and phosphoproteome sets.
**Figure S11:** Verification of transcript and protein levels related to major protein and starch biosynthesis in zmsnrk2.8;9;10 kernels at 15‐DAP.
**Figure 12**. Measurement and comparison of major SSREs activities in 15‐DAP kernels of zmsnrk2.8;9;10 and WT.
**Figure S13:** Phosphorylation sites of key SSREs and PPDKs based on phosphoproteomic analysis.
**Figure S14:** SSREs and PPDKs interact with ZmSnRK2.10 in maize kernels.
**Figure S15:** Subcellular localization and co‐localization of Bt1 and ZmSnRK2.10 using immunofluorescence.
**Figure S16:** Direct phosphorylation of BT1 by ZmSnRK2.8.
**Figure S17:** Phosphorylation sites and architecture of the Bt1 protein.
**Figure S18:** Identification of *Bt1*‐overexpression (*Bt1*‐OE) and *Bt1* ^*S141D*^‐overexpression (*Bt1* ^*S141D*^‐OE) lines in KN5585 background.
**Figure S19:** The interaction, phosphorylation, and subcellular localization of SSREs and ZmSnRK2.10.
**Figure S20:** Venn diagram showing the overlap of the 1290 TFs expressed in maize kernel (Chen et al. 2014) and DEGs, DAPs and DDPs in z*msnrk2.8;9;10*.
**Figure S21:** Temporal phosphoproteomic profiling and kinase prediction for O2 protein.
**Figure S22:** SDS‐PAGEs of the purified bacterially expressed recombinant proteins of O2, O2^S210AS212A^ and O2^S210DS212D^.
**Figure S23:** The transcriptional activity analysis of O2, O2^S210A212A^, and O2^S210D212D^.
**Figure S24:** The dynamic accumulation of substances in developing maize kernels.
**Figure S25:** Expression pattern analysis of *ZmSnRK2.10* using immunofluorescence.
**Figure S26:** Abscisic acid (ABA) and sucrose activate ZmSnRK2s protein kinase in maize kernels.
**Figure S27:** ABA concentration in WT and *zmsugcar1* kernels at 15‐DAP.
**Figure S28:** Altered expression levels of zein genes after treatment with sucrose, glucose, ABA, and sucrose plus ABA.
**Figure S29:** Identification of *ZmSnRK2.10*‐overexpression lines in B104 background.
**Figure S30:** Maize kernel length, kernel width and kernel thickness of F_1_ hybrids.

**Tables:** pbi70487‐sup‐0002‐TablesS1‐S8.rar.

## Data Availability

The raw data of RNA‐Seq analysis generated for this study have been submitted to the National Center for Biotechnology Information (NCBI) database under accession numbers PRJNA1226960. Accession Numbers: ZmSnRK2.8 (Zm00001d034161); ZmSnRK2.9 (Zm00001d033339); ZmSnRK2.10 (Zm00001d013736); ZmSnRK2.12 (Zm00001d013201). Sequence data from this article can be found in the MaizeGDB (https://maizegdb.org) database under the accession number.
