# Supplementary material for: Subclass III SnRK2 Kinases Coordinate Starch and Storage Protein Synthesis During Maize Kernel Development
Source: Plant Biotechnol J. 2025 Dec 8;24(4):2279–99. doi: 10.1111/pbi.70487 (PMC13140326; doi:10.1111/pbi.70487)
Supplement: Supplementary file 1 — Figure S1: CRISPR‐Cas9‐edited sequence of the ZmSnRK2.8/9/10/12 mutants. Figure S2: Phenotypic analysis of the WT and zmsnrk2s mutants. Figure S3: Kernel weights and major storage compound contents of WT and zmsnrk2 mutants. Figure S4. THE qRT‐PCR analysis of the expression profiles of subclass III ZmSnRK2 genes in developing maize kernels. Figure S5: Measurement of dry weight during developing kernels of WT and zmsnrk2.8;9;10. Figure S6: Paraffin sections of 10‐, 12‐, and 15‐DAP kernels of WT and zmsnrk2.8;9;10. Figure S7: Metabolome analysis of 15‐DAP WT and zmsnrk2.8;9;10 kernels. Figure S8: Characteristics of the omics data analyses. Figure S9: Characteristics of the comparative phosphoproteome in WT and zmsnrk2.8;9;10 filling kernels. Figure 10. Comparison and relationship of transcriptome, proteome, and phosphoproteome sets. Figure S11: Verification of transcript and protein levels related to major protein and starch biosynthesis in zmsnrk2.8;9;10 kernels at 15‐DAP. Figure 12. Measurement and comparison of major SSREs activities in 15‐DAP kernels of zmsnrk2.8;9;10 and WT. Figure S13: Phosphorylation sites of key SSREs and PPDKs based on phosphoproteomic analysis. Figure S14: SSREs and PPDKs interact with ZmSnRK2.10 in maize kernels. Figure S15: Subcellular localization and co‐localization of Bt1 and ZmSnRK2.10 using immunofluorescence. Figure S16: Direct phosphorylation of BT1 by ZmSnRK2.8. Figure S17: Phosphorylation sites and architecture of the Bt1 protein. Figure S18: Identification of Bt1‐overexpression (Bt1‐OE) and Bt1 S141D ‐overexpression (Bt1 S141D ‐OE) lines in KN5585 background. Figure S19: The interaction, phosphorylation, and subcellular localization of SSREs and ZmSnRK2.10. Figure S20: Venn diagram showing the overlap of the 1290 TFs expressed in maize kernel (Chen et al. 2014) and DEGs, DAPs and DDPs in zmsnrk2.8;9;10. Figure S21: Temporal phosphoproteomic profiling and kinase prediction for O2 protein. Figure S22: SDS‐PAGEs of the purified bact [file PBI-24-2279-s002.docx]

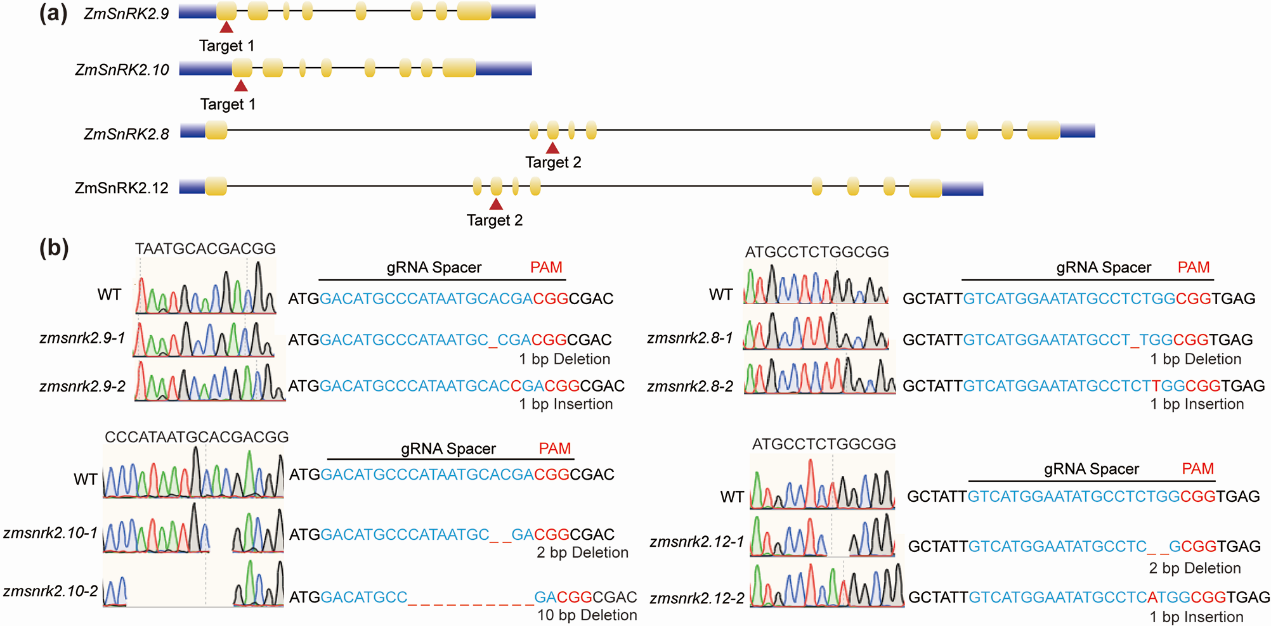


**Supplemental Figure 1. CRISPR-Cas9-edited sequence of the *ZmSnRK2.8/9/10/12* mutants.**

**(a)** The gene structure of subclass III ZmSnRK2s and the positions of the gRNAs. Yellow rectangles indicate exons. Blue rectangles indicate 5’ and 3’ UTR. The red triangles indicate the gRNA target sites in the knockout constructs. **(b)** Detailed DNA sequence edits of two independent mutant lines for *ZmSnRK2.8*, *ZmSnRK2.9*, *ZmSnRK2.10,* and *ZmSnRK2.12* in KN5585 background. PAM, protospacer adjacent motif.


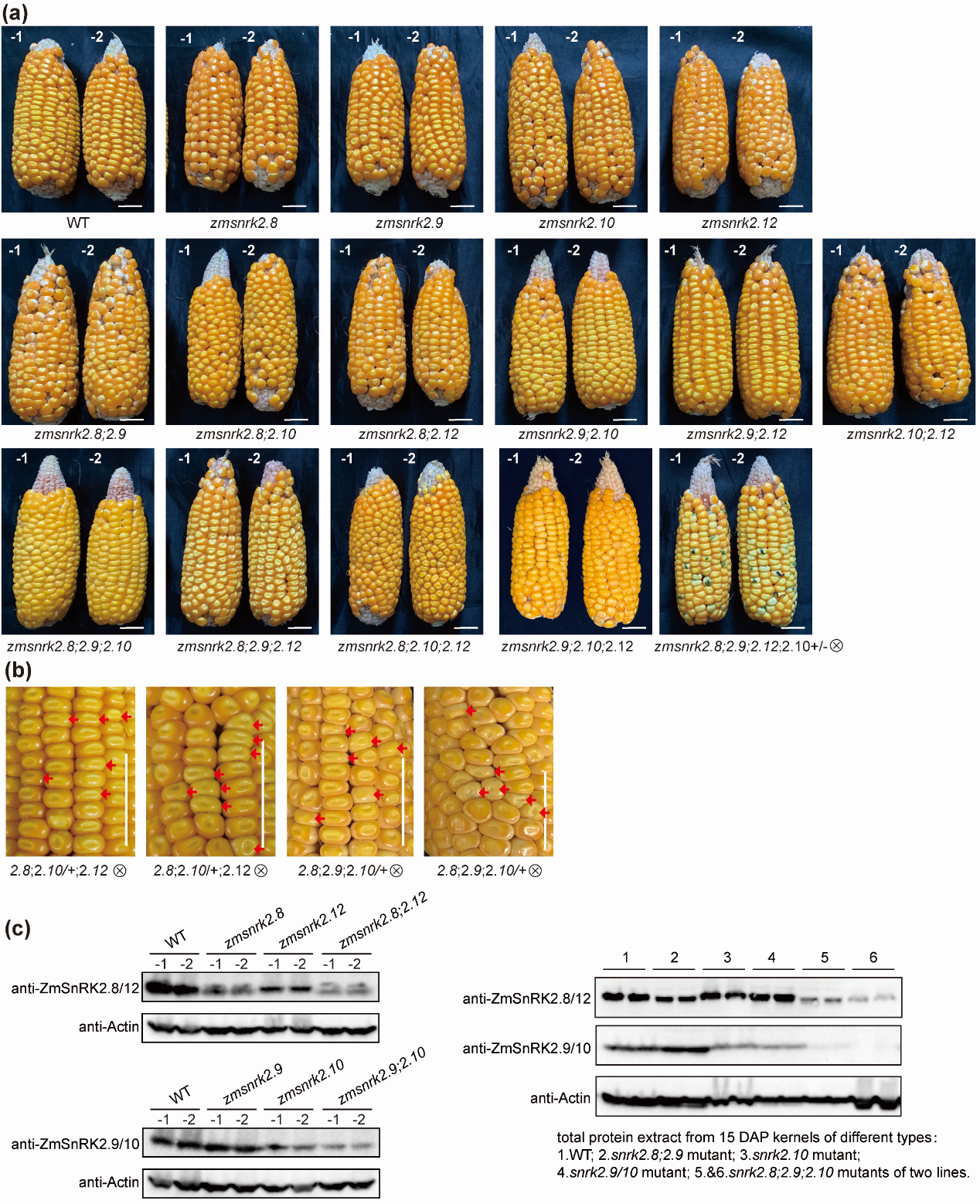


**Supplemental Figure 2. Phenotypic analysis of the WT and *zmsnrk2s* mutants.**

**(a)** The ear phenotypes of the WT and different *zmsnrk2s* mutant lines. Scale bar, 3 cm. **(b)** The ear phenotypes of different heterozygotes. Scale bar, 3 cm. **(c)** Immunoblot analysis of protein levels of ZmSnRK2s in 15-DAP kernels of the WT and different *znsnrk2s* mutant lines. Each sample was detected with anti-ZmSnRK2.8/12 and/or anti-ZmSnRK2.9/10. Actin was used as an internal control.


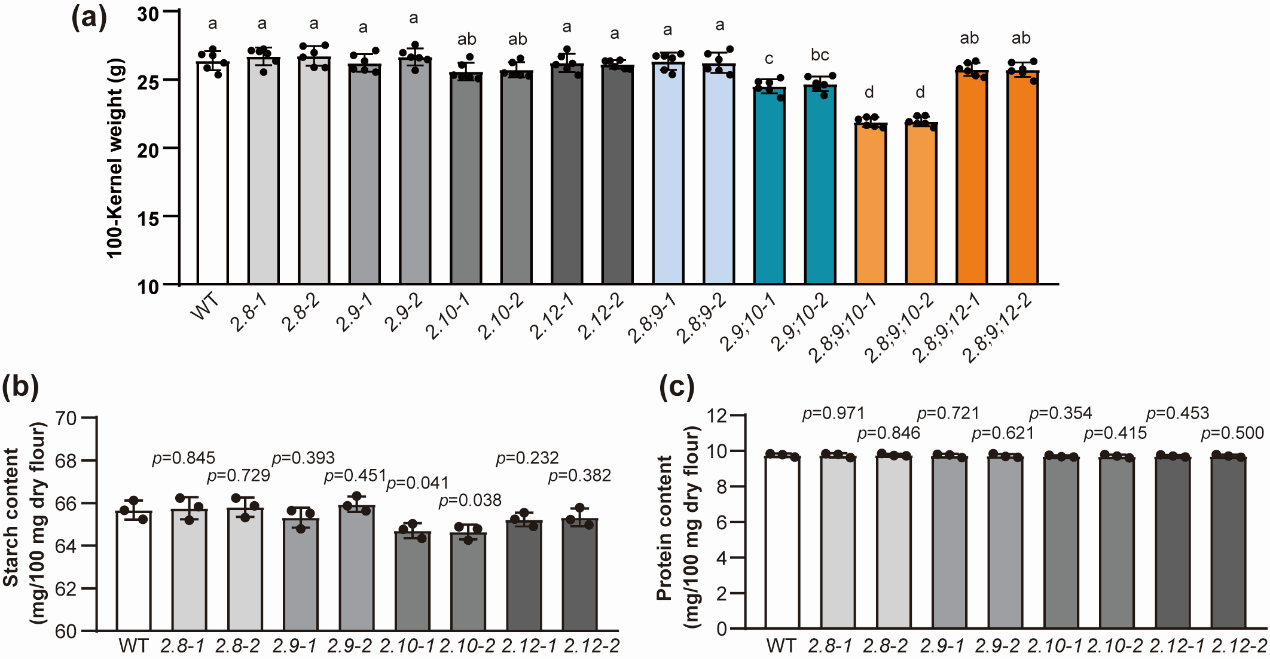


**Supplemental Figure 3. Kernel weights and major storage compound contents of WT and *zmsnrk2* mutants.**

**(a)** Measurement of 100-kernel weight of different genotypes of *zmsnrk2s* knockout mutants. Data are means ± SD (n=6; different letters indicate significant differences; *P* < 0.05, Duncan multiple range test for each interval). **(b,c)** Measurement of starch (**b**) and protein (**c**) contents in mature kernels of WT and all the single-mutant lines of subclass III ZmSnRK2s. Data are means ± SD (n=3). Statistical significance was determined by two-tailed Student’s *t*-test.


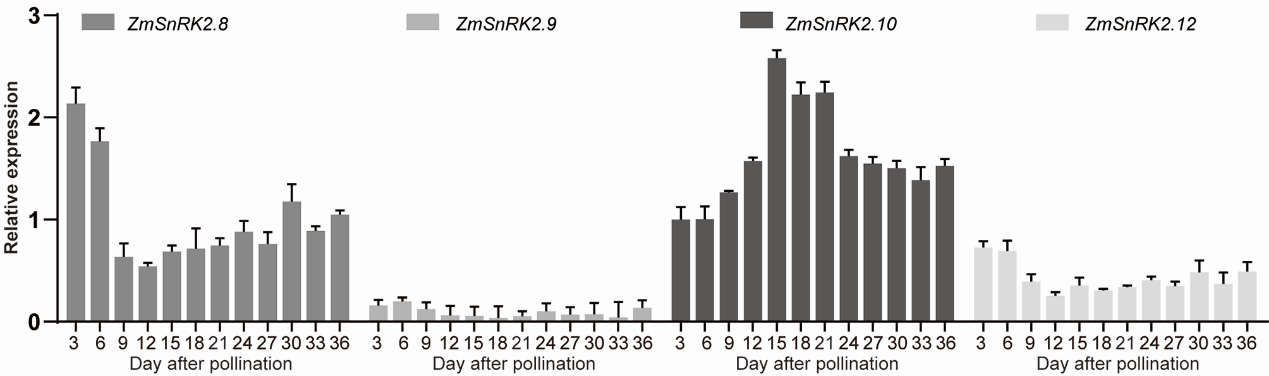


**Supplementary Figure 4. The qRT-PCR analysis of the expression profiles of subclass III *ZmSnRK2* genes in developing maize kernels.** All expression levels were normalized to *ZmActin*. Data are means ± SD (n=3).


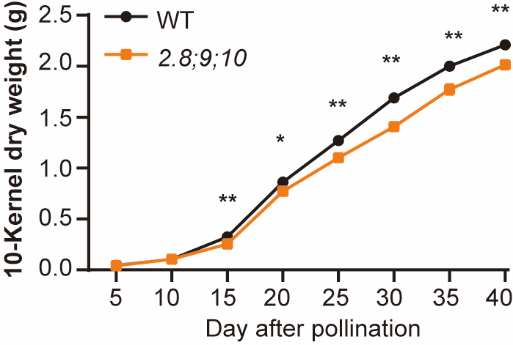


**Supplemental Figure 5. Measurement of dry weight during developing kernels of WT and *zmsnrk2.8;9;10.***

Data are means ± SD (n=5). Statistical significance (**P* < 0.05; ***P* < 0.01) was determined by two-tailed Student’s *t*-test.


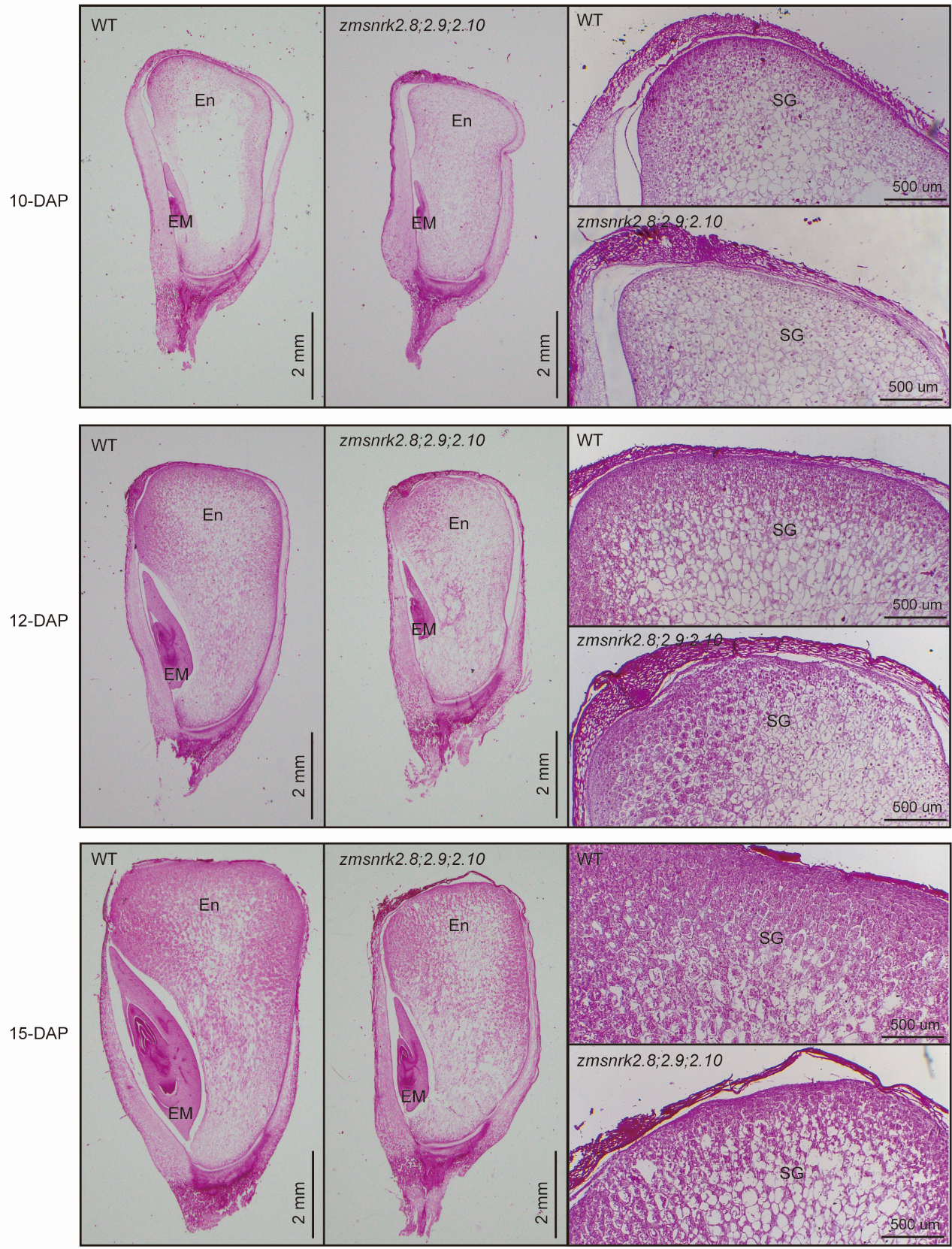


**Supplemental Figure 6. Paraffin sections of 10-, 12- and 15-DAP kernels of WT and *zmsnrk2.8;9;10*.**

En: endosperm; EM: embryo; SG: starch granule.


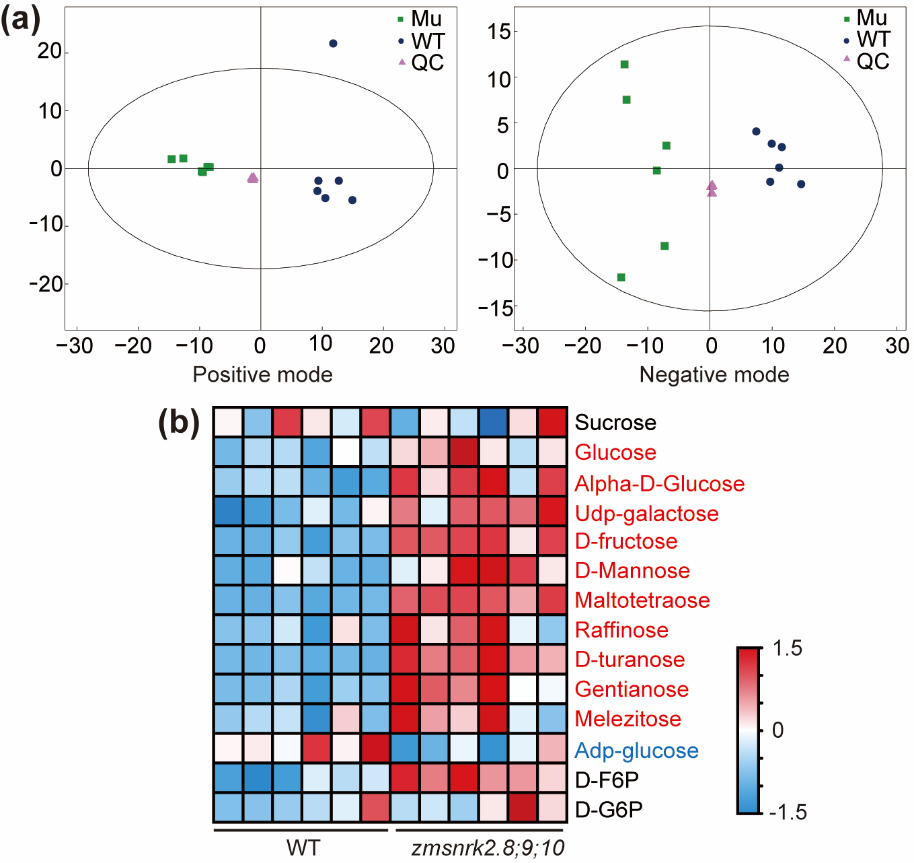


**Supplemental Figure 7.** **Metabolome analysis of 15-DAP WT and *zmsnrk2.8;9;10* kernels.**

**(a)** Principal component analyses (PCA) of nontargeted metabolomes. Metabolites were extracted from six individual samples of 15-DAP kernels and analyzed in both positive and negative models. **(b)** Relative saccharide content in *zmsnrk2.8;9;10* compared to WT. Metabolites shown in red, blue and black indicate significantly increased, significantly decreased, and unchanged levels in *zmsnrk2.8;9;10*, respectively.


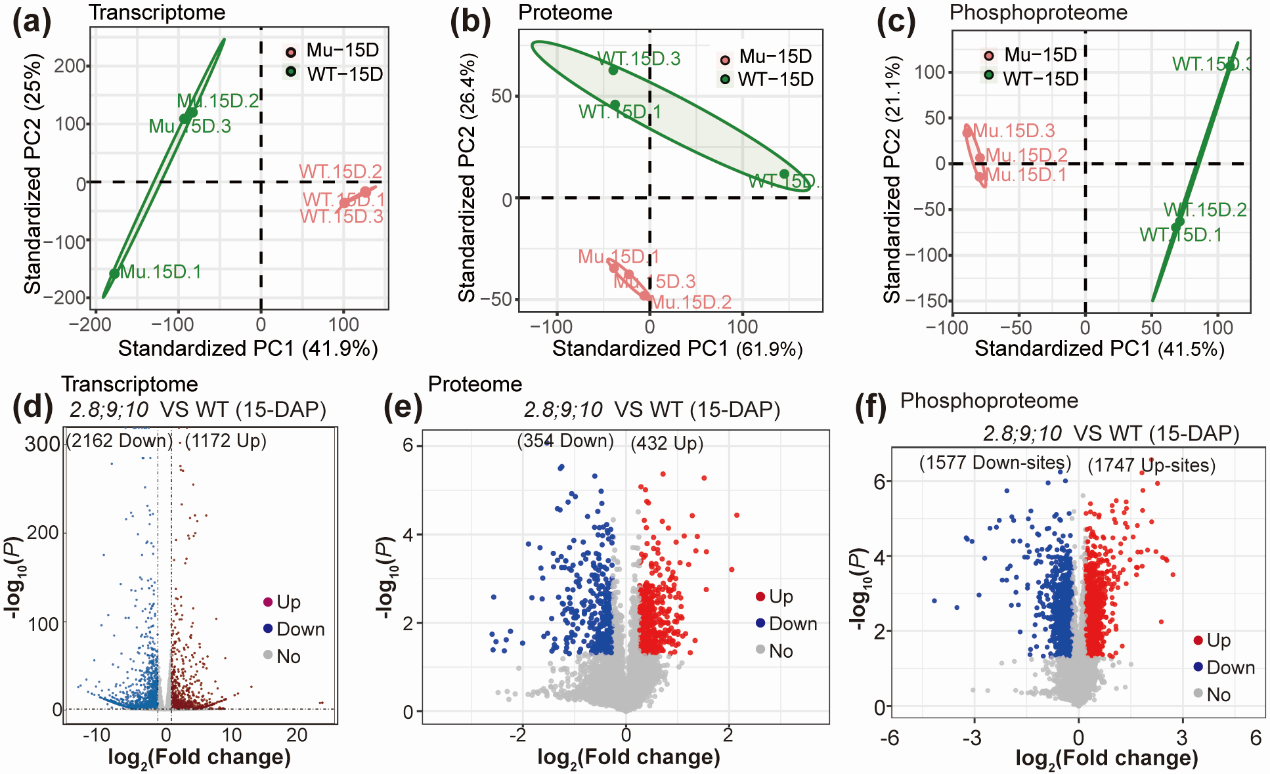


**Supplemental Figure 8. Characteristics of the omics data analyses.**

**(a-c)** Principal component analyses of transcriptome (**a**), proteome (**b**), and phosphoproteome (**c**) data sets, respectively. **(d-f)** Volcano plots showing the numbers of differentially expressed genes (DEGs) (**d**), differentially abundant proteins (DAPs) (**e**), and differentially phosphorylated proteins (DPPs) (**f**) between WT and *zmsnrk2.8;9;10* kernels at 15-DAP. Red, blue, and gray dots indicate significantly upregulated, significantly downregulated, and unchanged genes or proteins, respectively.


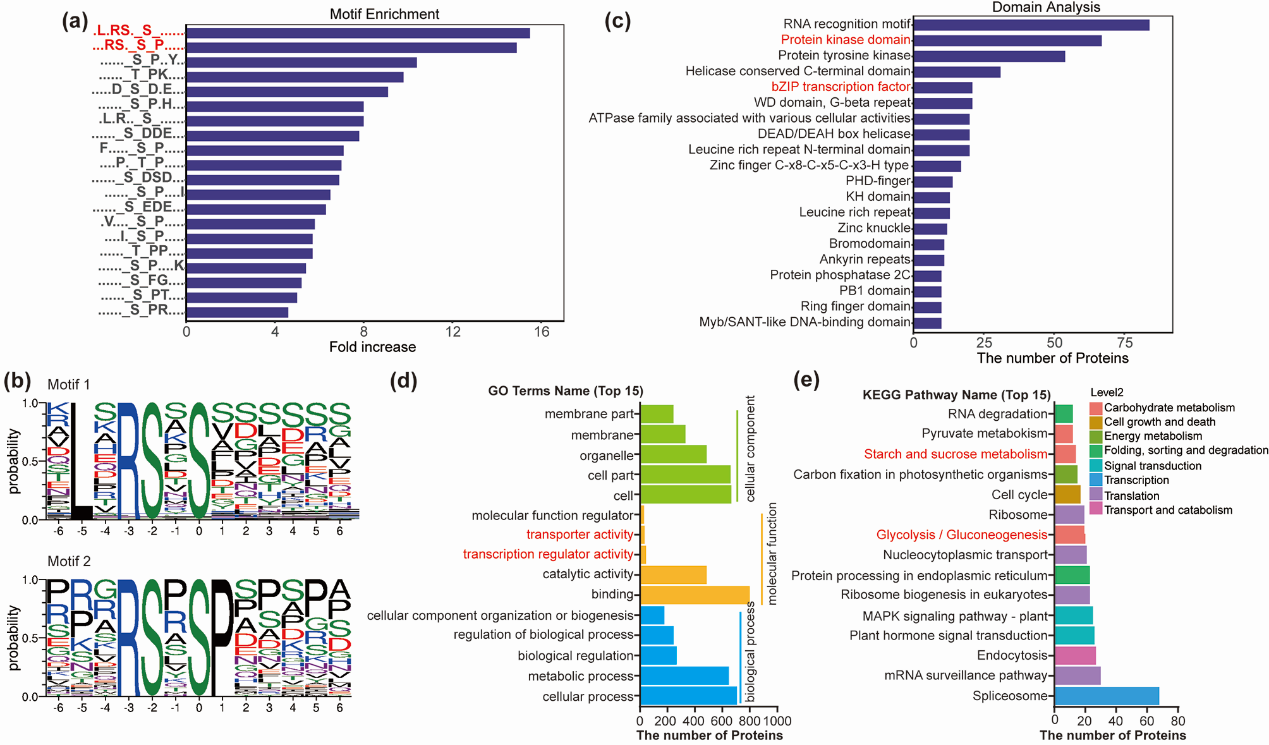


**Supplemental Figure 9. Characteristics of the comparative phosphoproteome in WT and z*msnrk2.8;9;10* filling kernels.**

**(a)** The major motifs identified from the putative substrates of subclass III ZmSnRK2s. **(b)** Top two significantly enriched motifs were obtained through the motif analysis. **(c)** Domain analysis of the putative substrates of subclass III ZmSnRK2s. **(d,e)** Gene ontology (GO) term (**d**) and kyoto encyclopedia of genes and genomes (KEGG) pathway (**e**) statistics of the DPPs.


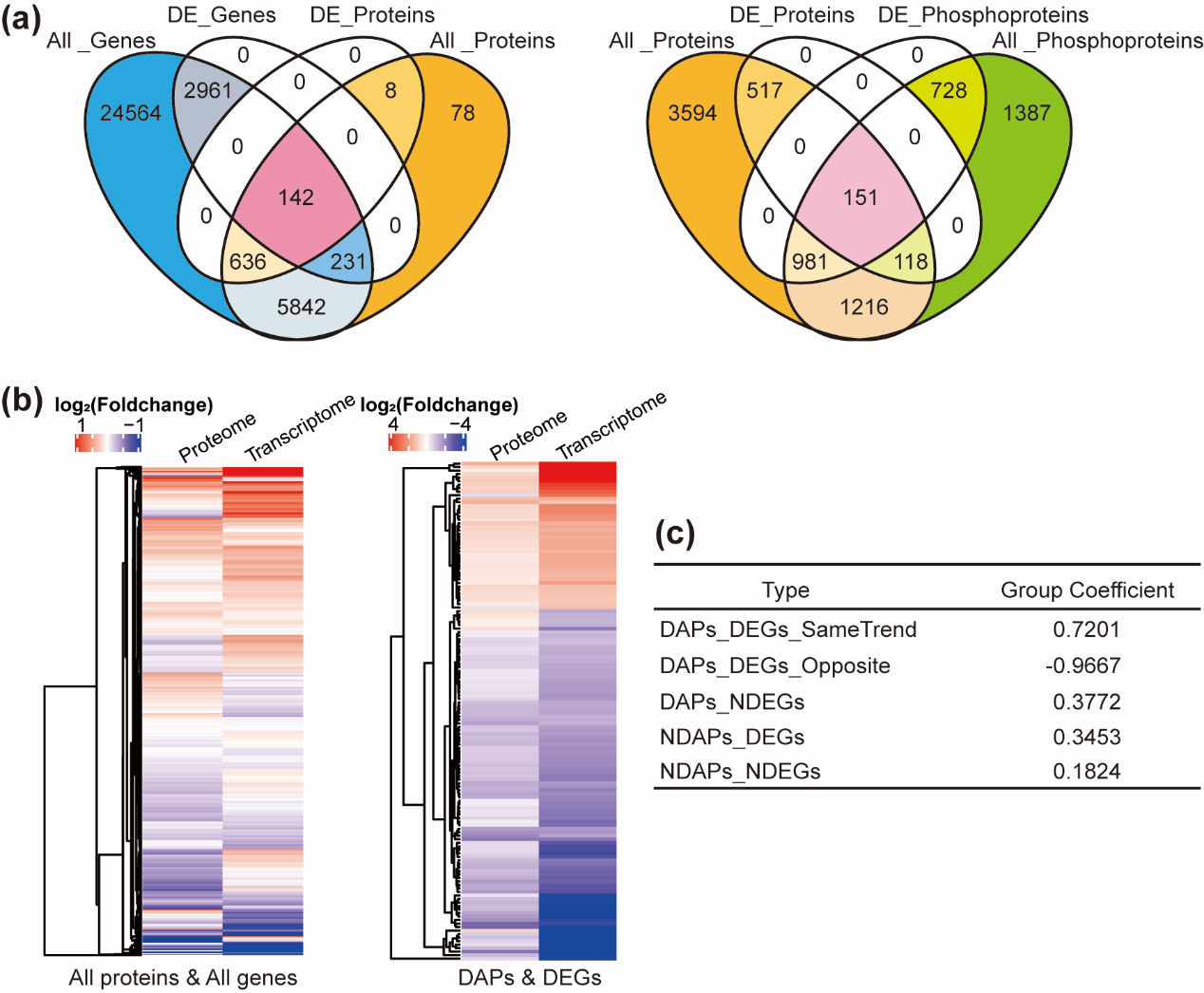


**Supplemental Figure 10. Comparison and relationship of transcriptome, proteome, and phosphoproteome sets.**

**(a)** Venn diagram showing the relationship between DEGs and DAPs, and the relationship between DAPs and DPPs. **(b)** Hierarchical clustering of all genes identified in both the transcriptome and proteome (left), and hierarchical clustering showing the DAPs and DEGs in association (right). The color indicates the value of log_2_(fold change). Red indicates that the protein/gene expression was up-regulated in the sample, and blue indicates that the expression was down regulated. **(c)** Summary of the correlation coefficient between genes identified in both the transcriptome and proteome. DAPs_DEGs_SameTrend: mRNA and protein levels of DAPs and DEGs showed the same trend. DAPs_DEGs_Opposite: mRNA and protein levels of DAPs and DEGs showed the opposite trend. DAPs_NDEGs: there were differences in protein expression and no differences in mRNA expression. NDAPs_DEGs: there were no differences in protein expression and differences in mRNA expression. NDAPs_DEGs: there were no differences in protein expression and mRNA expression.


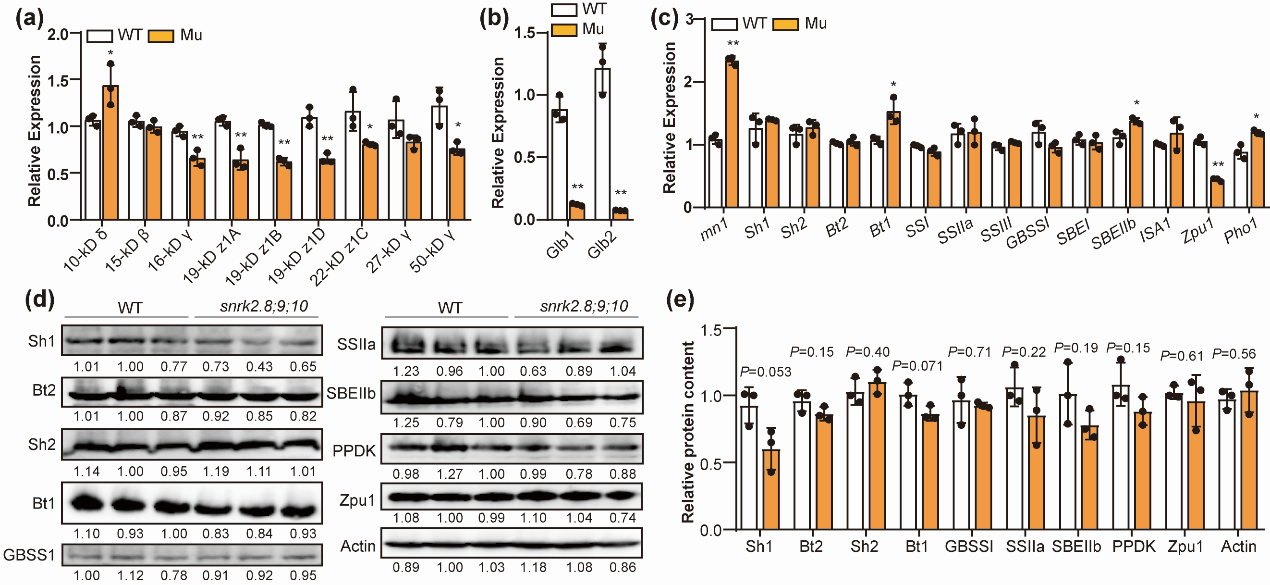


**Supplemental Figure 11. Verification of transcript and protein levels related to major protein and starch biosynthesis in *zmsnrk2.8;9;10* kernels at 15-DAP.**

**(a, b)** qRT-PCR analysis of genes encoding major zeins (**a**) and globulins (**b**). Data are means ± SD (n=3). **(c)** qRT-PCR analysis of genes encoding starch synthesis-related enzymes (SSREs). Data are means ± SD (n=3) **(d, e)** Immunoblot analysis of major SSREs and PPDKs in 15-DAP kernels of *zmsnrk2.8;9;10* and WT (**d**). Actin was used as an internal control. Three independent samples were analyzed for each material, and the protein bands were quantified using the ImageJ software, as shown in (**e**). Data are means ± SD (n=3). Statistical significance (**P < 0.05*; ***P < 0.01*) was determined by two-tailed Student’s *t*-test, as shown in (**a-c, e**).


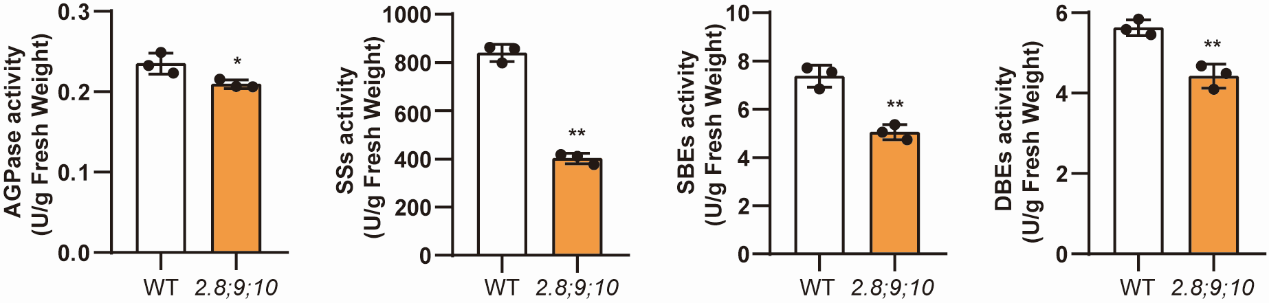


**Supplemental Figure 12. Measurement and comparison of major SSREs activities in 15-DAP kernels of *zmsnrk2.8;9;10* and WT.**

Data are means ± SD (n=3). Statistical significance (**P <* 0.05; ***P <* 0.01) was determined by two-tailed Student’s *t*-test.


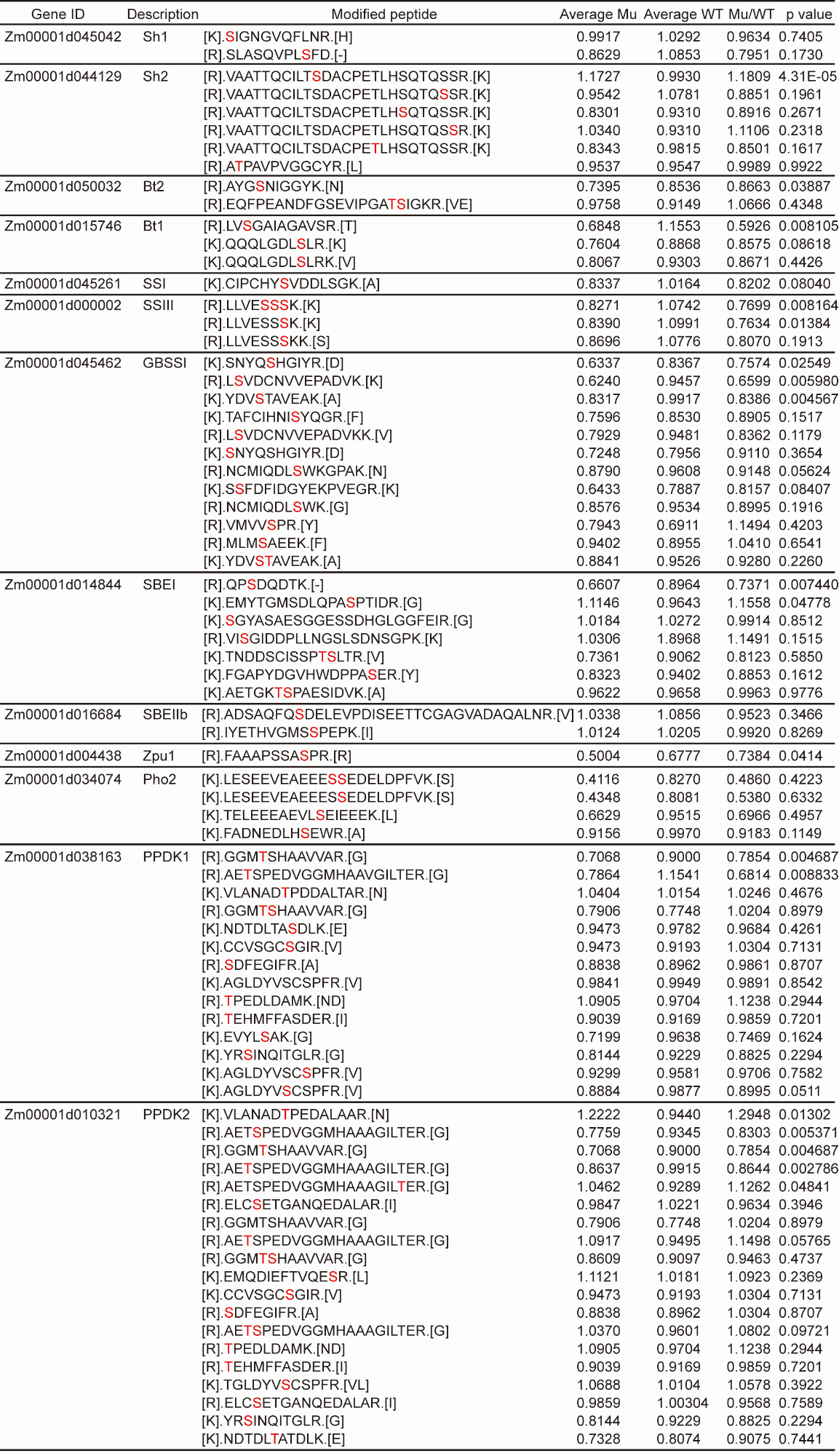


**Supplementary Figure 13. Phosphorylation sites of key SSREs and PPDKs based on phosphoproteomic analysis.**


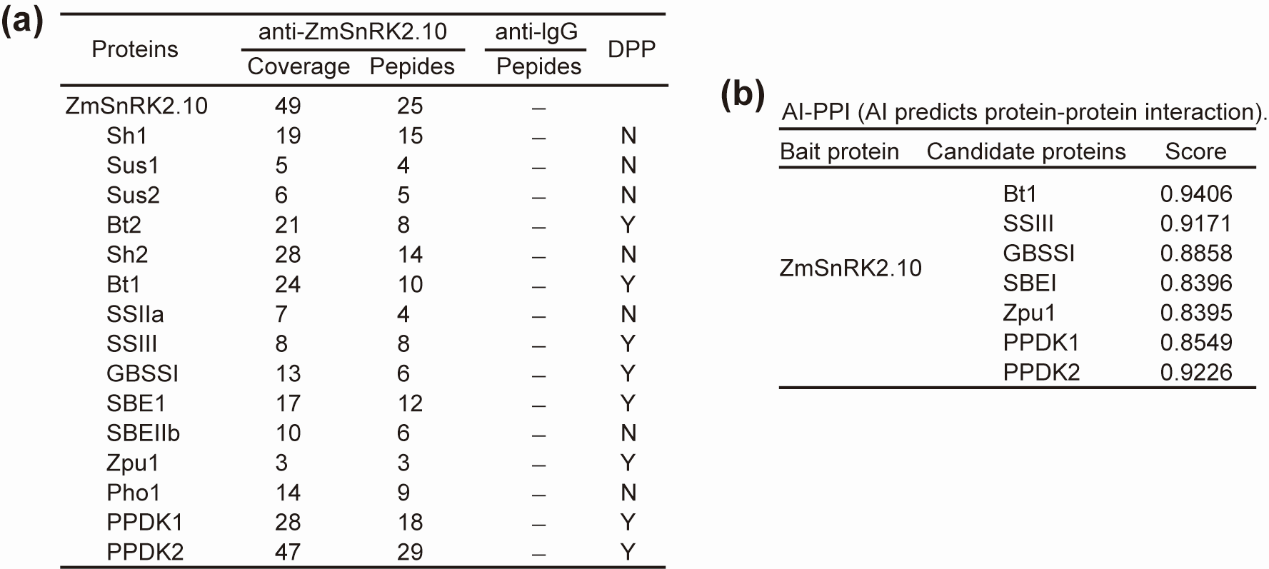


**Supplementary Figure 14. SSREs and PPDKs interact with ZmSnRK2.10 in maize kernels.**

**(a)** List of SSREs and PPDKs interacting with ZmSnRK2.10, identified by immunoprecipitation mass spectrometry (IP-MS) using an anti-ZmSnRK2.10 antibody. DPP, differentially phosphorylated protein; N, no; Y, yes. **(b)** List of SSREs and PPDKs interacting with ZmSnRK2.10, predicted by AI-PPI (artificial intelligence–based prediction of protein-protein interactions).

**
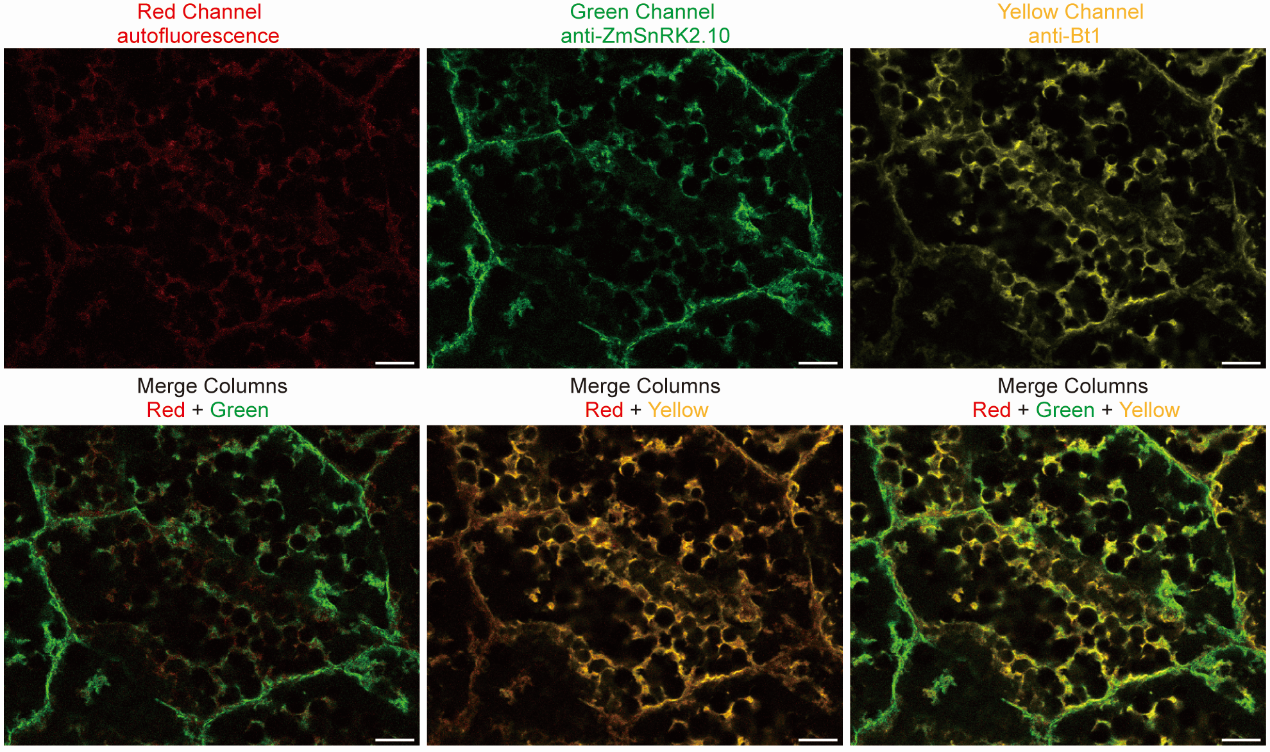
**

**Supplementary Figure 15.** **Subcellular localization and co-localization of Bt1 and ZmSnRK2.10 using immunofluorescence.**

Immunofluorescence analysis of ZmSnRK2.10 and Bt1 protein accumulation patterns in maize endosperm at 15-DAP. Representative micrographs were obtained through scanning electron microscopy (SEM) imaging. Immunodetection was conducted using **anti-Bt1 and anti-ZmSnRK2.10 antibodies.** Scale bars, 20 μm.

**
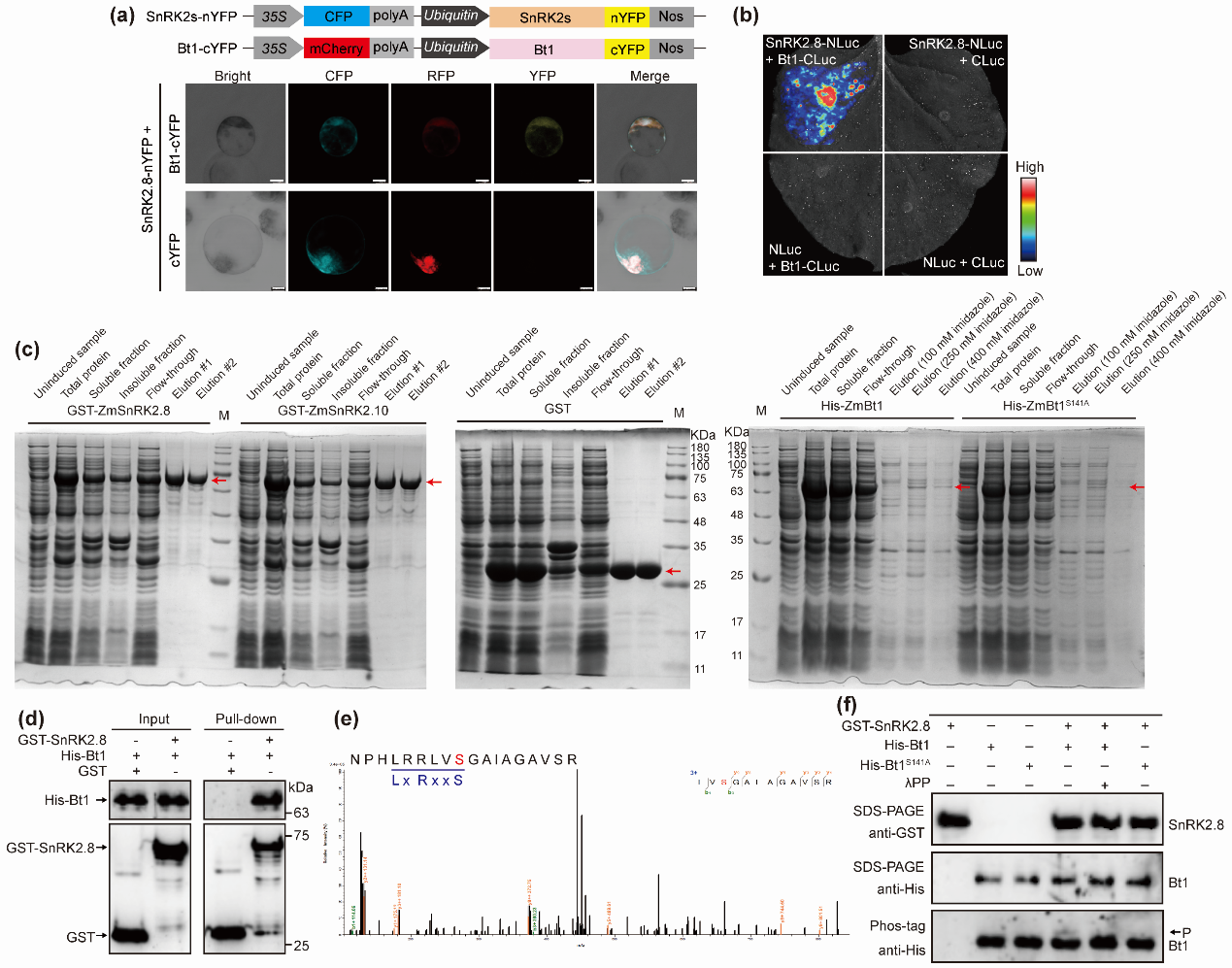
**

**Supplementary Figure 16.** **Direct phosphorylation of BT1 by ZmSnRK2.8.**

**(a)** BiFC assay showing the interaction between ZmSnRK2.8 with BT1 in maize leaf protoplasts. Scale bars, 10 μm. CFP, cyan fluorescent protein. YFP, yellow fluorescent protein. **(b)** LCI assay showing the interaction between ZmSnRK2.8 with BT1 in *Nicotiana benthamiana* leaves. High, strong LUC intensity; Low, weak LUC intensity. **(c)** SDS-PAGEs of the purified bacterially expressed recombinant proteins of ZmSnRK2.8, ZmSnRK2.10, Bt1 and Bt1^S141A^. All constructs were expressed in *E. coli* Rosetta (DE3) cells and purified using corresponding resin and reagents. M, marker. The red arrows indicate the corresponding target protein bands. **(d)** GST pull-down assay showing the interaction between ZmSnRK2.8 with BT1. **(e)** Identification of Ser141 in the Bt1 protein phosphorylated by subclass III ZmSnRK2s using liquid chromatography-tandem mass spectrometry (LC-MS/MS). Phosphorylated amino acids in the identified peptides were marked in red. The LxRxxS motif, recognized primarily by SnRK2s, was marked in blue. **(f)** *In vitro* kinase assay showing phosphorylation modification of BT1 by ZmSnRK2.8 and identification of phosphorylation sites. Lambda protein phosphatase (λPP) was used to dephosphorylate the induced phosphorylation proteins. The slow migrated band indicated the phosphorylated BT1 protein in the Phos-tag gel and is marked with the arrow.

**
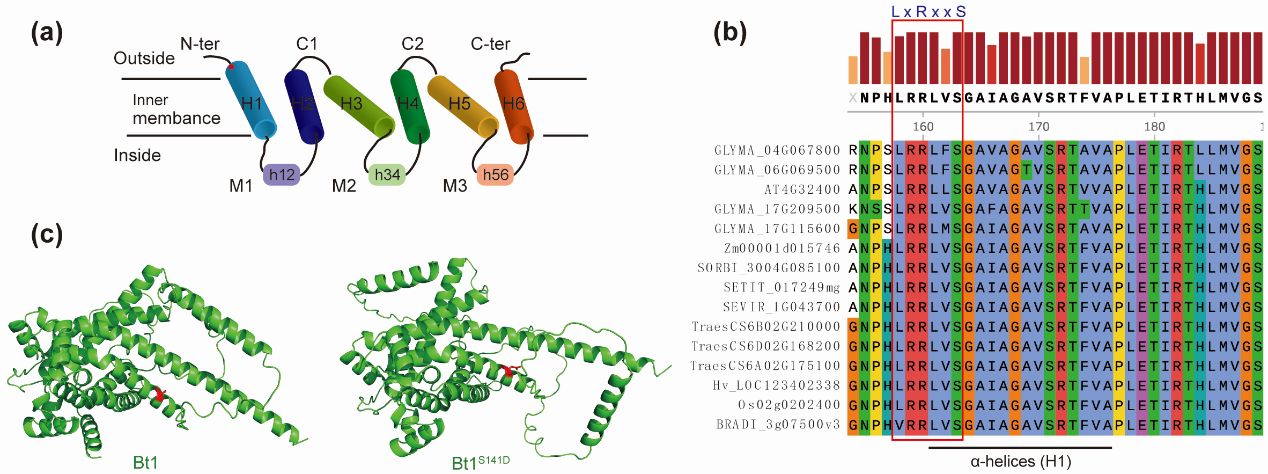
**

**Supplementary Figure 17. Phosphorylation sites and architecture of the Bt1 protein.**

**(a)** Schematic diagram showing the architecture of the Bt1 protein. Transmembrane helices, surface helices, intermembrane space loops, and matrix loops are labelled H, h, C, and M, respectively. Helices comprised the following residues: 139–154 (H1), 193–210 (H2), 233–249 (H3), 286–303 (H4), 331–346 (H5), 389–406 (H6). S141 is marked by a red dot. Inside and outside were designated the amyloplast and cytosol, respectively. **(b)** Amino acid sequence alignment of Bt1 and its homologs in plants. The homologous proteins of Bt1 were obtained via NCBI blast (<https://www.ncbi.nlm.nih.gov/)>. **(c)** Structural model of Bt1 and Bt1^S141D^ complex predicted by AlphaFold 3. The phosphorylation site of Bt1-S141 was marked in red.

**
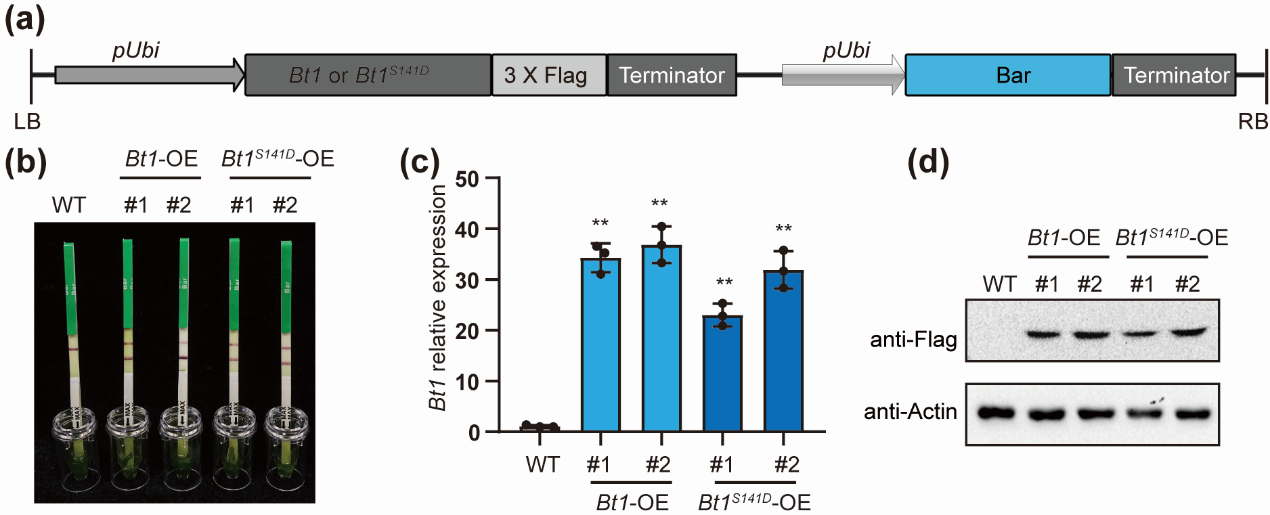
**

**Supplementary Figure 18. Identification of *Bt1*-overexpression (*Bt1*-OE) and *Bt1^S141D^*-overexpression (*Bt1^S141D^*-OE) lines in KN5585 background.**

**(a)** Schematic diagram of *Bt1-Flag* and *Bt1^S141D^-Flag* overexpression vector. **(b)** Identification of *Bt1*-OE and *Bt1^S141D^*-OE lines using PAT/bar quick strips. **(c)** qRT-PCR analysis of *Bt1* expression in 15-DAP kernels of the WT, *Bt1*-OE and *Bt1^S141D^*-OE lines. Data are means ± SD (n=3). Statistical significance (***P* < 0.01) was determined by two-tailed Student’s *t*-test. **(d)** The protein level of Flag in 15-DAP kernels from WT, *Bt1*-OE and *Bt1^S141D^*-OE lines. Actin was used as an internal control.

**
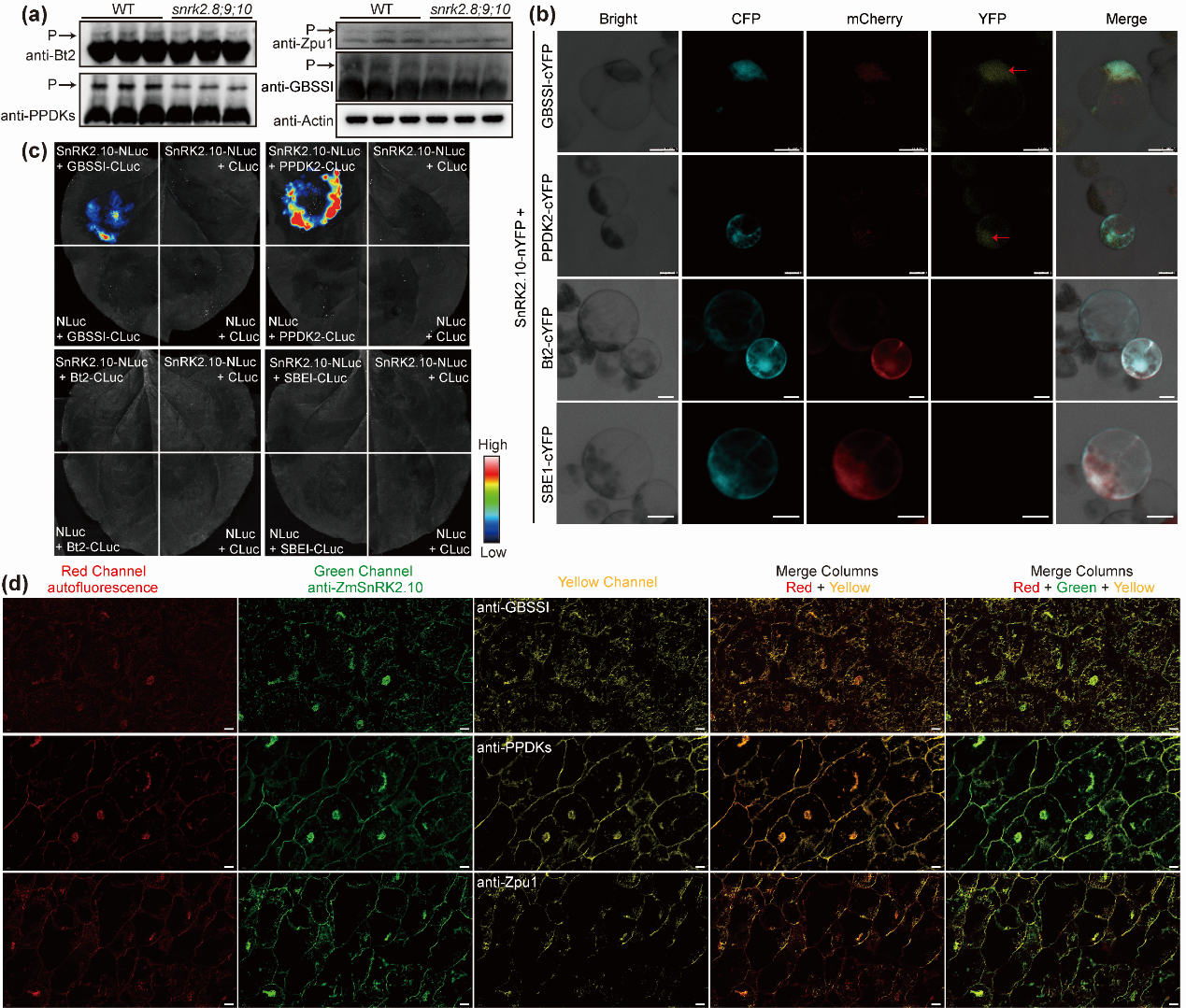
**

**Supplementary Figure 19. The interaction, phosphorylation and subcellular localization of SSREs and ZmSnRK2.10.**

**(a)** The phosphorylation levels of Bt2, PPDKs, Zpu1 and GBSSI in *zmsnrk2.8;9;10* and WT kernels. The slow migrated band indicated the phosphorylated proteins in the Phos-tag gel and is marked with the arrow. **(b)** BiFC assay showing that ZmSnRK2.10 interacts with PPDK2 and GBSSI, but not with Bt2 or SBE1, in maize leaf protoplasts. Scale bars, 10 μm. CFP, cyan fluorescent protein. YFP, yellow fluorescent protein. **(c)** LCI assay showing that ZmSnRK2.10 interacts with PPDK2 and GBSSI, but not with Bt2 or SBE1 in *Nicotiana benthamiana* leaves. High, strong LUC intensity; Low, weak LUC intensity. **(d)** Subcellular localization and co-localization of GBSSI, PPDKs, Zpu1 and ZmSnRK2.10 using immunofluorescence in 15-DAP maize endosperm. Representative micrographs were obtained through scanning electron microscopy (SEM) imaging. Immunodetection was conducted using **anti-**GBSSI, **anti-**PPDKs, **anti-**Zpu1 **and anti-ZmSnRK2.10 antibodies.** Scale bars, 20 μm.

**
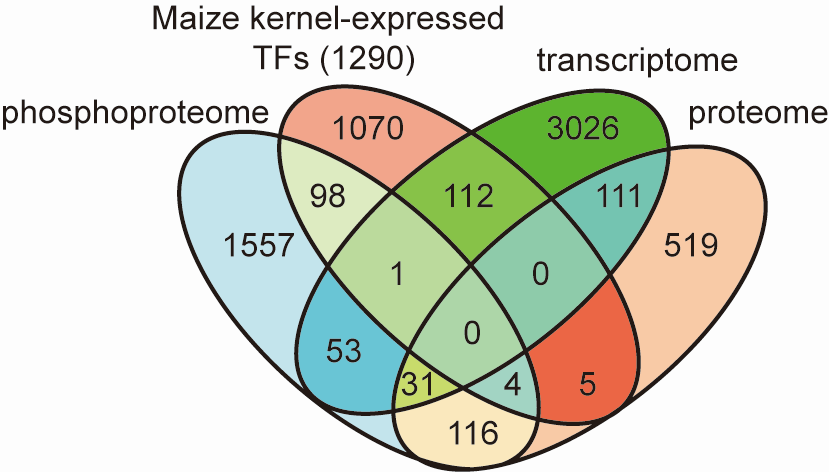
**

**Supplementary Figure 20. Venn diagram showing the overlap of the 1,290 TFs expressed in maize kernel (Chen *et al.*, 2014) and DEGs, DAPs and DDPs in z*msnrk2.8;9;10*.**

**
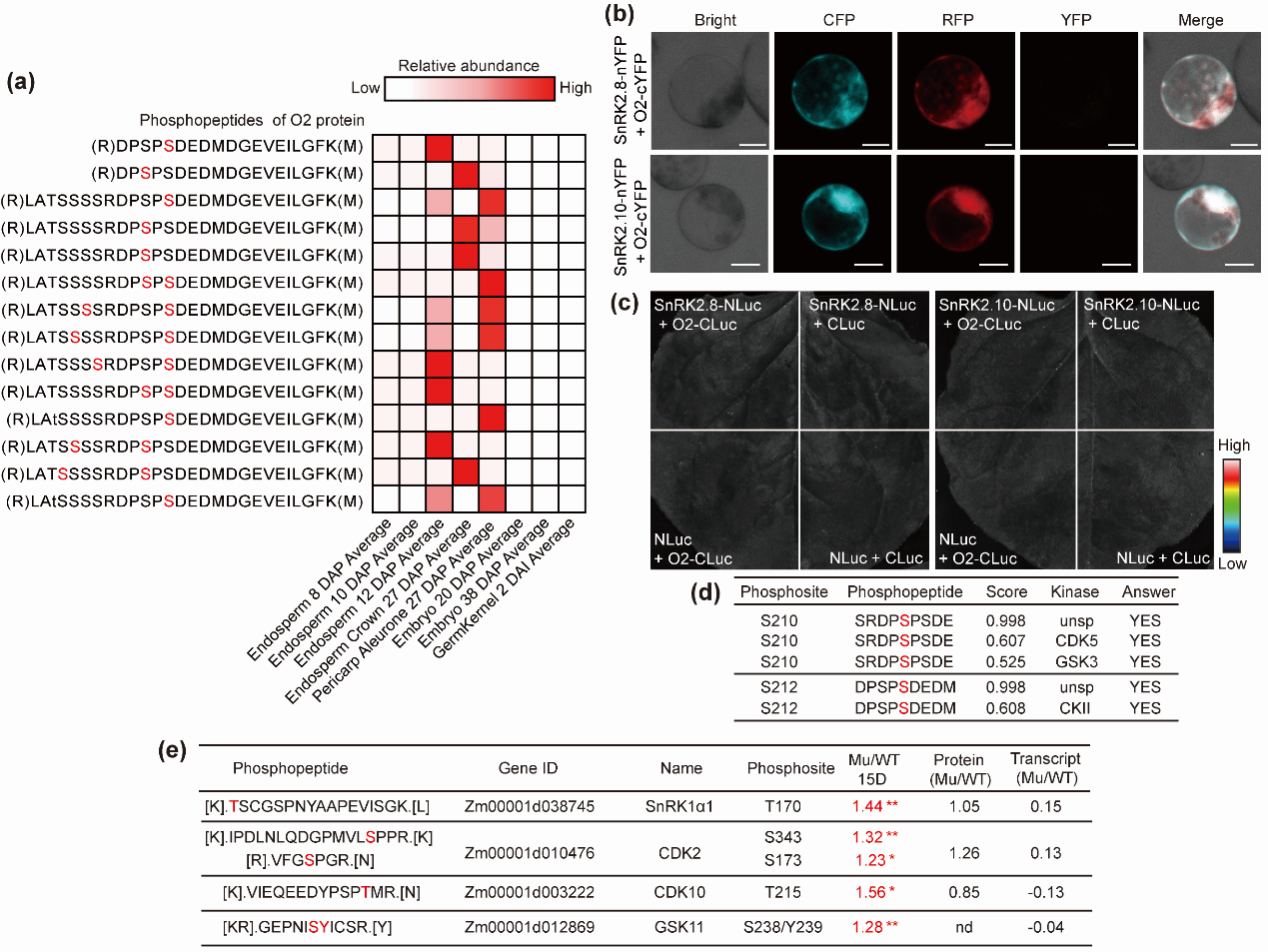
**

**Supplementary Figure 21. Temporal phosphoproteomic profiling and kinase prediction for O2 protein.**

**(a)** Heat maps depict the relative abundance of all phosphopeptides of O2 protein throughout kernel development. Color gradient reflects normalized intensity values (log_2_-transformed) of identified phosphorylation sites (Ser/Thr) across developmental timepoints. The source data were downloaded from Walley *et al.*, (2013). **(b)** BiFC assays showing no interaction between ZmSnRK2.8 or ZmSnRK2.10 and O2 in maize leaf protoplasts. Scale bars, 10 μm. CFP, cyan fluorescent protein. YFP, yellow fluorescent protein. **(c)** LCI assay showing no interaction between ZmSnRK2.8 or ZmSnRK2.10 and O2 in *Nicotiana benthamiana* leaves. High, strong LUC intensity; Low, weak LUC intensity. **(d)** Predicted protein kinases targeting the O2 S210/212 phosphopeptide. Kinase prediction was performed using NetPhos 3.1. Scores > 0.5 indicate positive predictions (“YES”). “unsp” denotes non-specific predictions where the kinase could not be assigned to a known kinase family. **(e)** List of potential kinases of O2 identified among subclass III ZmSnRK2s dependent phosphoproteins, including gene IDs, phosphopeptide(s), phosphosites, and phosphorylation changes in transcription and protein levels in z*msnrk2.8;9;10* compared to WT.


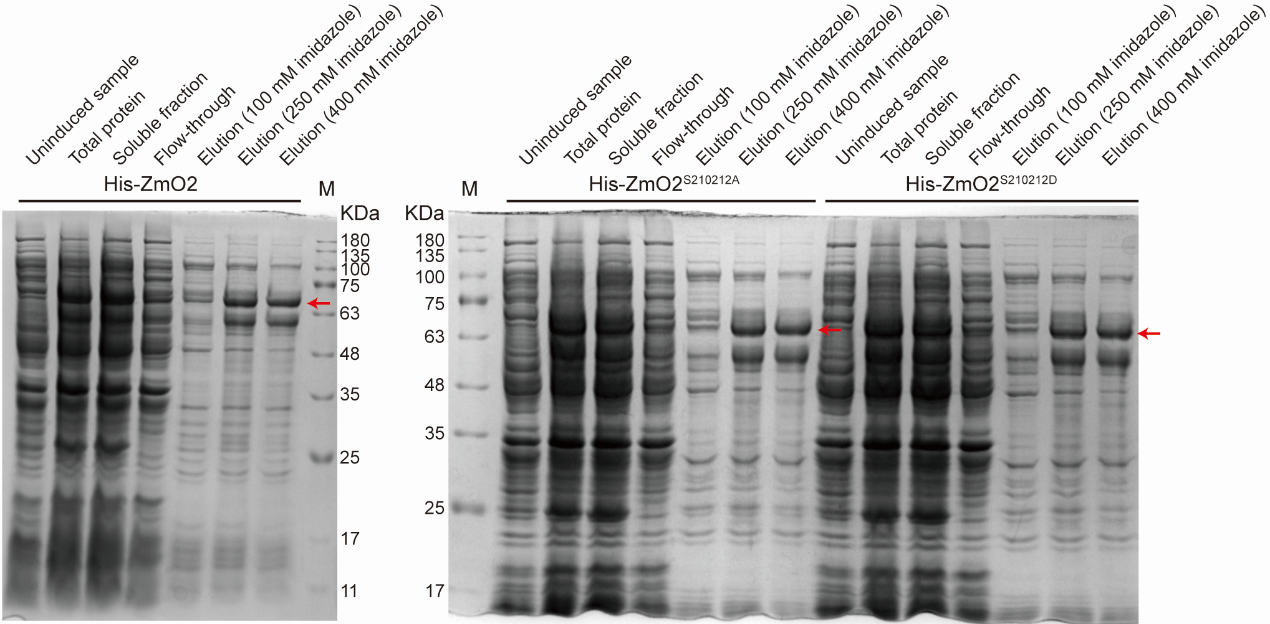


**Supplementary Figure 22.** **SDS-PAGEs of the purified bacterially expressed recombinant proteins of O2, O2^S210AS212A^ and O2^S210DS212D^.**

All constructs were expressed in *E. coli* Rosetta (DE3) cells and purified using His-tag resin and gradient concentrations of imidazole. M, marker. The red arrows indicate the corresponding target protein bands.

**
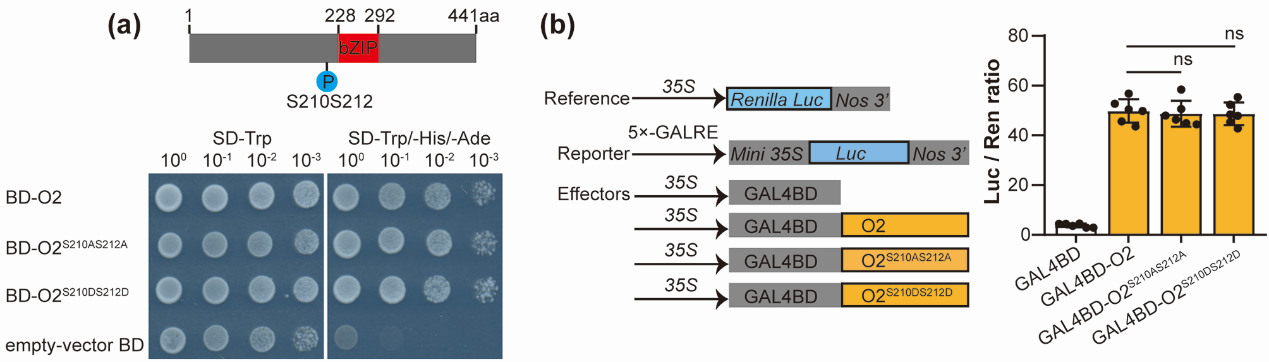
**

**Supplementary Figure 23. The transcriptional activity analysis of O2, O2^S210A212A^, and O2^S210D212D^.**

**(a)** Yeast transcriptional activity analysis of O2, O2^S210A212A^, and O2^S210D212D^. The schematic showed the O2 protein architecture and its major phosphorylation sites based on the phosphoproteomic data. The O2, O2^S210A212A^, and O2^S210D212D^ were cloned into the pGBKT7 vector and expressed in the AH109 yeast strain. Transactivation activity was monitored by growth on SD−Trp/−His/-Ade plates. The empty vector pGBKT7 served as a negative control. **(b)** The transcriptional activities of O2, O2^S210A212A^, and O2^S210D212D^ were detected in maize leaf protoplasts. The left panel is a vector construction diagram. LUC, firefly luciferase; 35S, CaMV 35S promoter; Ren, Renilla Luc. The ratio of Luc/Ren in the right panel represents the activity of O2, O2^S210A212A^, and O2^S210D212D^. Data are means ± SD (n=6). Statistical significance (ns, not significant) was determined by two-tailed Student’s *t*-test.


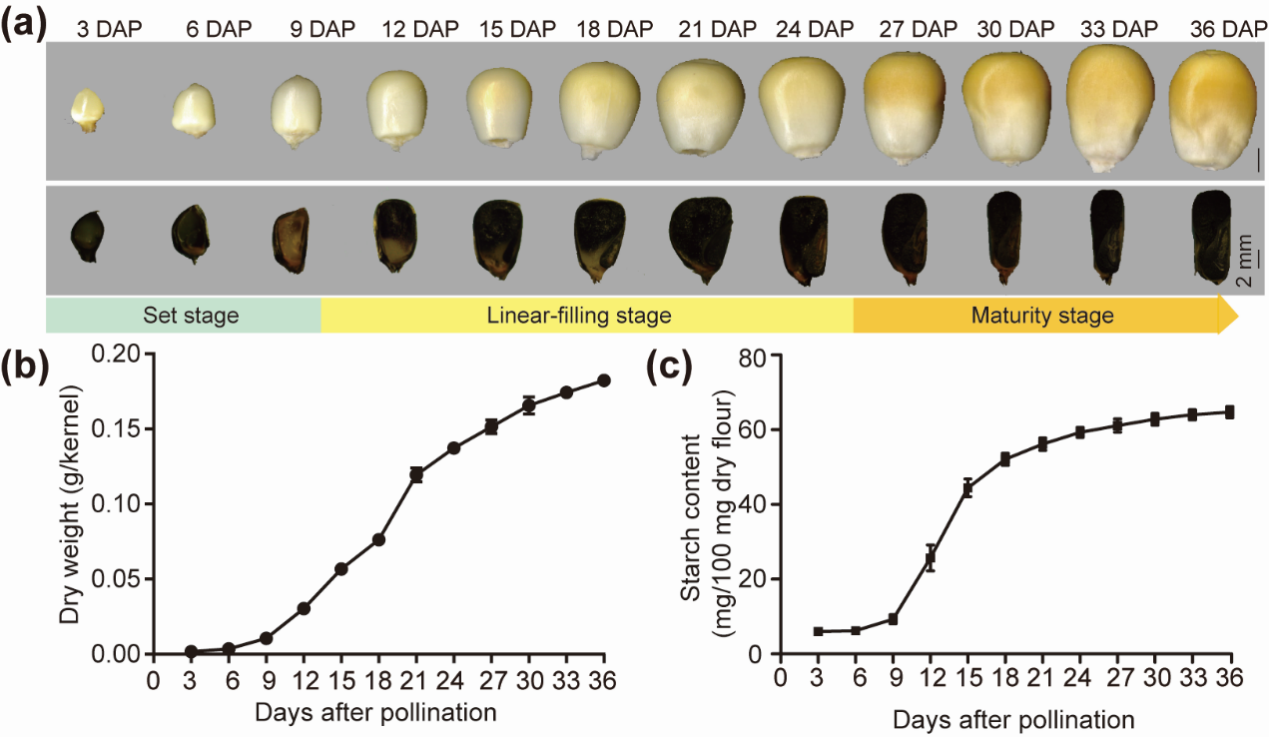


**Supplementary Figure 24. The dynamic accumulation of substances in developing maize kernels.**

**(a)** Photographs of developing B73 kernels stained with I_2_/KI on longitudinal sections. Scale bar, 2 mm. **(b, c)** Dry weight (**b**, n = 12) and starch content (**c**, n = 6) in maize developing kernels. Data are means ± SD.


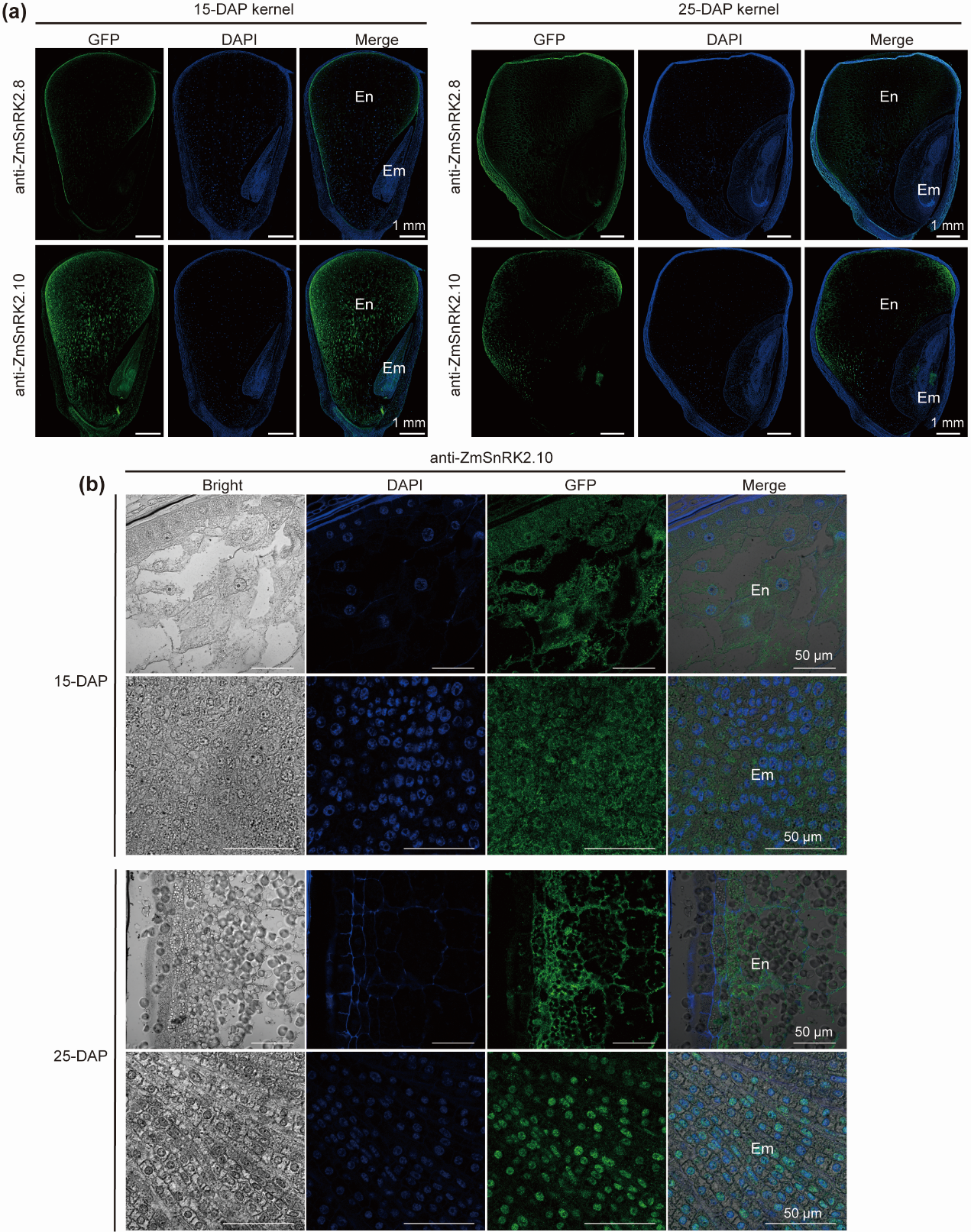


**Supplementary Figure 25. Expression pattern analysis of *ZmSnRK2.10* using immunofluorescence.**

**(a)** Immunofluorescence analysis of ZmSnRK2.8 and ZmSnRK2.10 protein accumulation patterns in developing maize kernels at 15 and 25 DAP, respectively. Representative micrographs were obtained through SEM imaging. Immunodetection was conducted using anti-**ZmSnRK2.8 and anti-ZmSnRK2.10 antibodies. (b)** Subcellular immunolocalization of ZmSnRK2.10 protein in developing endosperm and embryo (15 DAP and 25 DAP). High-resolution images were captured using laser scanning confocal microscopy (LSCM). Nuclei were counterstained with DAPI (4',6-diamidino-2-phenylindole). En, endosperm; Em, embryo.

**
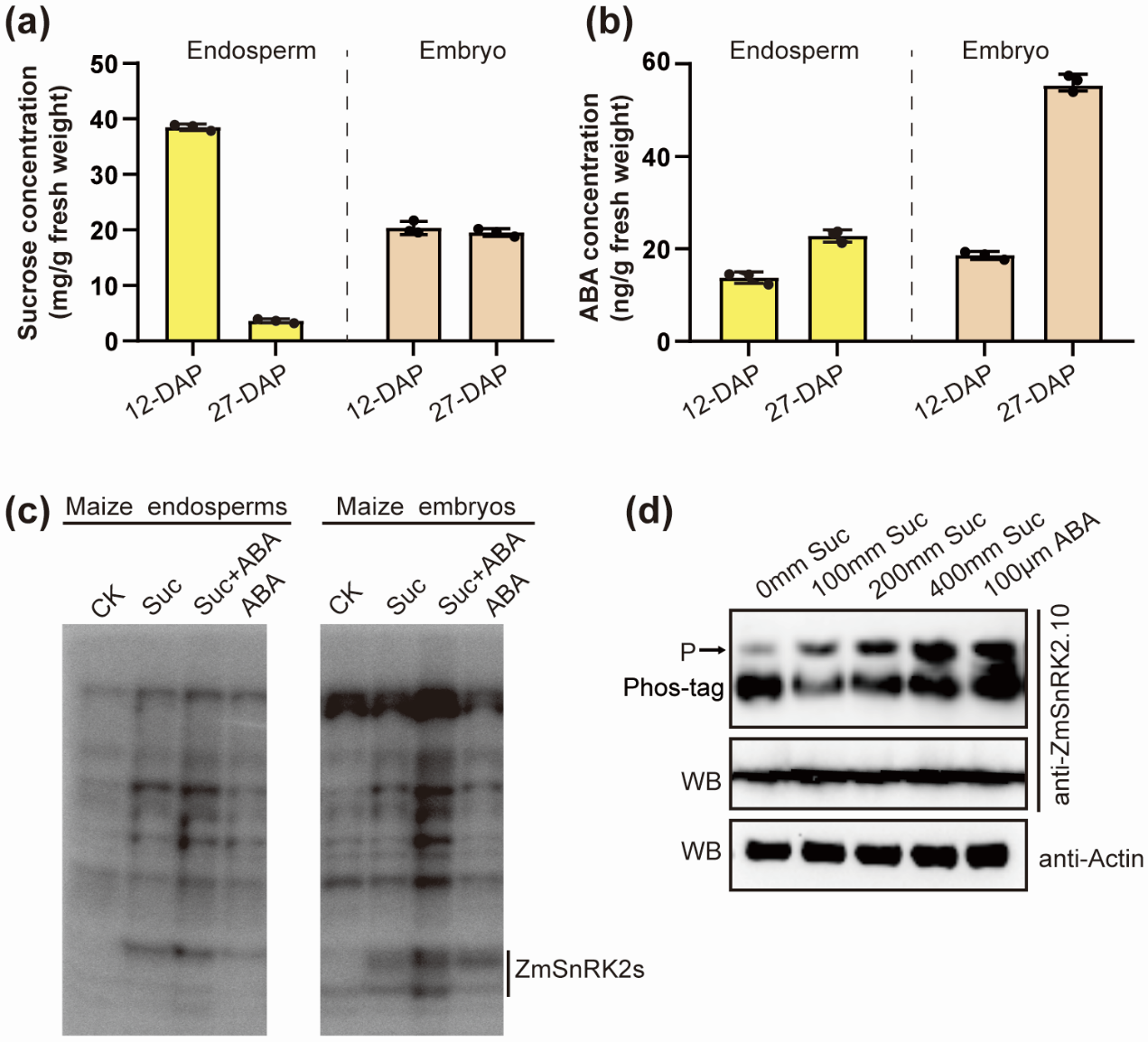
**

**Supplementary Figure 26. Abscisic acid (ABA) and sucrose activate ZmSnRK2s protein kinase in maize kernels.**

**(a)** Concentrations of sucrose in maize endosperm and embryo at 12 and 27-DAP. Data are means ± SD (n=3). **(b)** Concentrations of ABA in maize endosperm and embryo at 12 and 27-DAP. Data are means ± SD (n=3). **(c)** In-gel kinase assay showing ZmSnRK2s kinase activities in 15-DAP endosperms (left panel) and 15-DAP embryos (right panel) after treatment with sucrose, ABA, and sucrose plus ABA. Sucrose: 200 mM, ABA: 100 μM. The image is representative of two independent experiments. **(d)** Phos-tag SDS-PAGE showing the phosphorylation of ZmSnRK2.10 in 15-DAP kernels after sucrose and ABA treatment. The slow migrated band indicated the phosphorylated ZmSnRK2.10 protein in the Phos-tag gel and is marked with the arrow. Actin was used as an internal control.

**
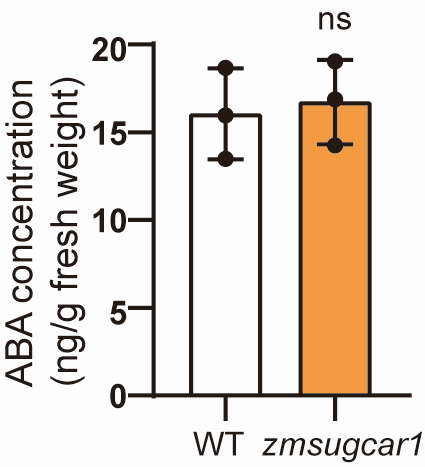
**

**Supplementary Figure 27. ABA concentration in WT and *zmsugcar1* kernels at 15-DAP.**

Data are means ± SD (n=3). Statistical significance was determined by two-tailed Student’s *t*-test. ns: not significant.

**
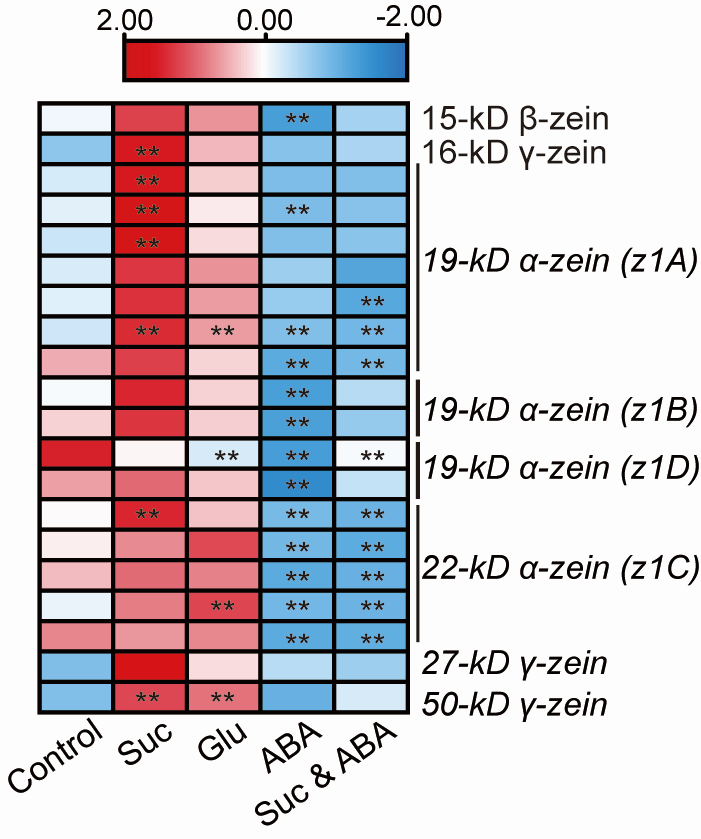
**

**Supplementary Figure 28. Altered expression levels of zein genes after treatment with sucrose, glucose, ABA, and sucrose plus ABA.**

Expression data were from our previous report (Huang *et al.*, 2016). Items with corrected *P* < 0.05 were considered DEGs, with *P*-values indicated by stars (***P* < 0.01).


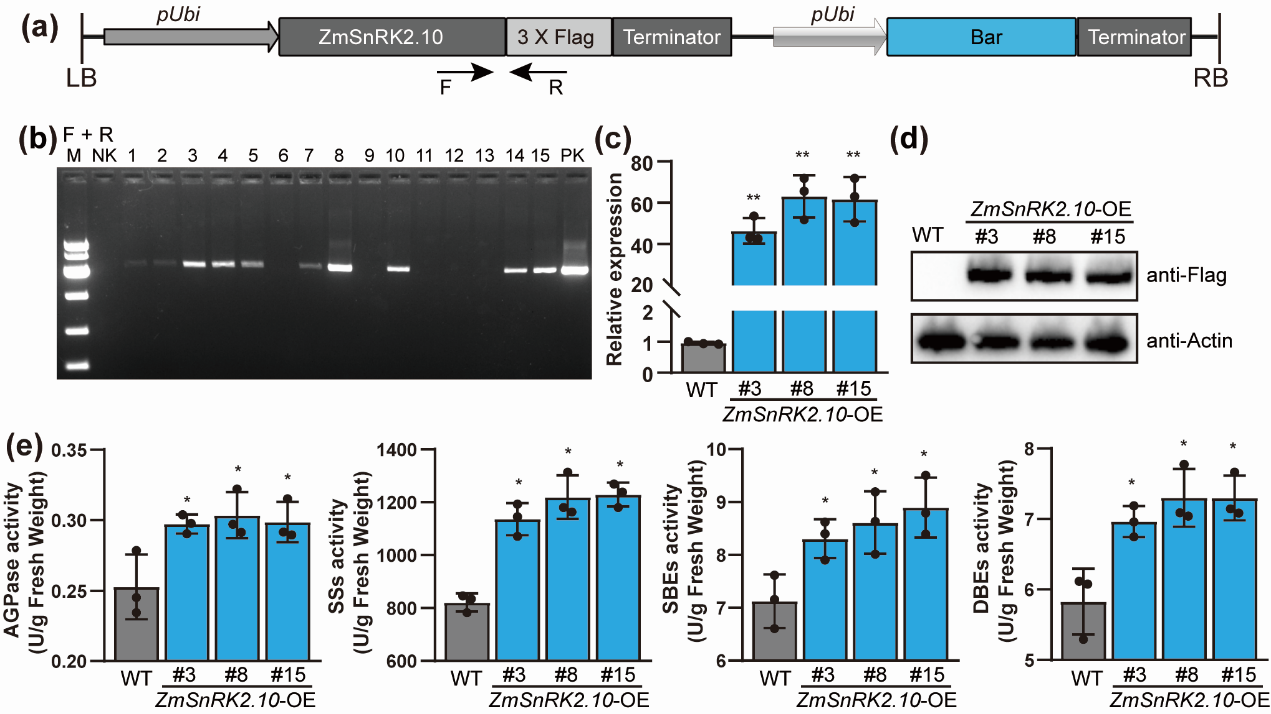


**Supplementary Figure 29. Identification of *ZmSnRK2.10*-overexpression lines in B104 background.**

**(a)** Schematic diagram of *ZmSnRK2.10-Flag* overexpression vector. **(b)** DNA amplification analysis identifying *ZmSnRK2.10*-overexpression lines. M, marker. NK, negative control. PK, positive control. **(c)** qRT-PCR analysis of *ZmSnRK2.10* expression in 15-DAP kernels of the WT and *ZmSnRK2.10*-OE lines (#3, #8, and #15). All expression levels were normalized to *ZmActin*. Data are means ± SD (n=3). **(d)** The protein level of ZmSnRK2.10 in 15-DAP kernels from WT and *ZmSnRK2.10*-OE lines. ZmSnRK2.10 was detected with anti-Flag. Actin was used as an internal control. **(e)** Measurement and comparison of the major starch biosynthetic enzymes activities in 15-DAP kernels from *ZmSnRK2.10*-OE lines and WT. Data are means ± SD (n=3). Statistical significance (**P <* 0.05; ***P <* 0.01) was determined by two-tailed Student’s *t*-test, as shown in (**c, e**).


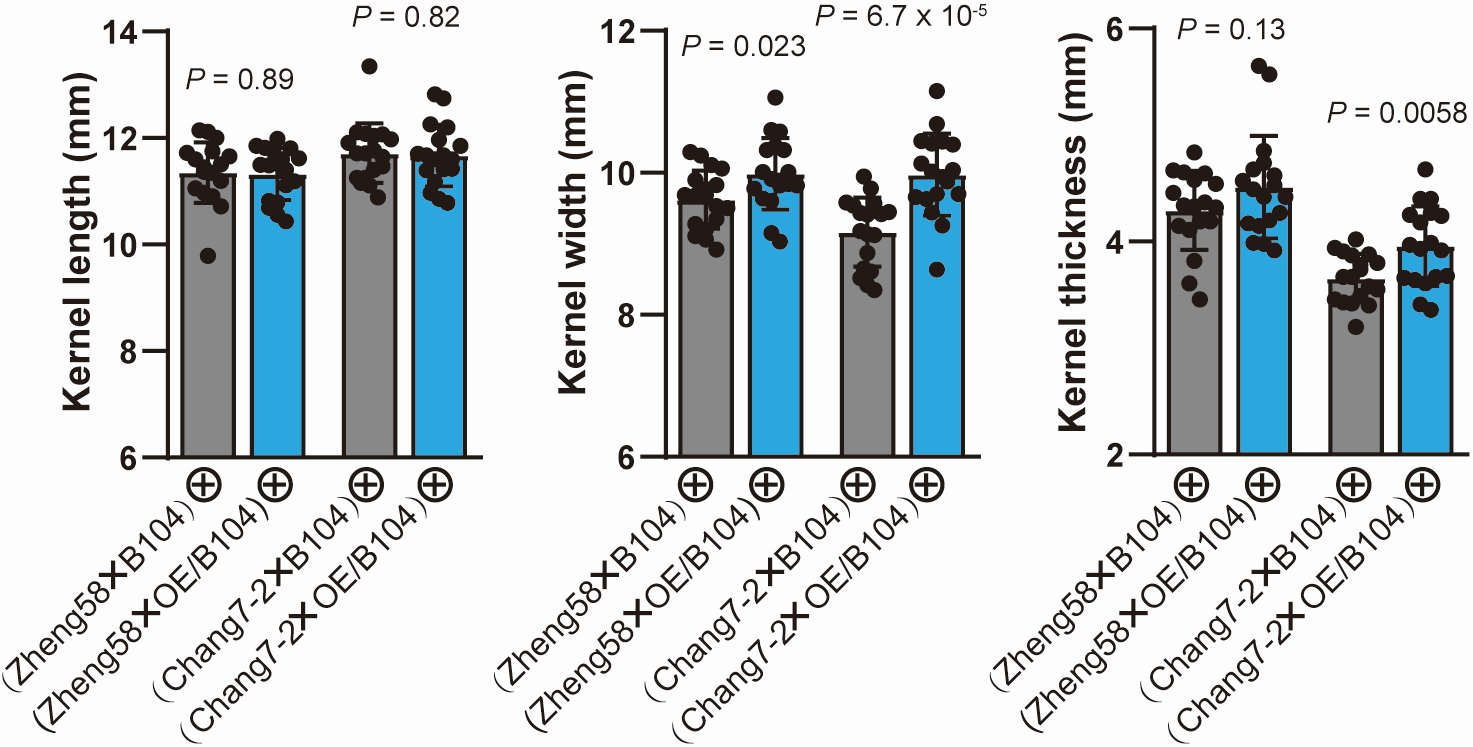


**Supplementary Figure 30. Maize kernel length, kernel width and kernel thickness of F_1_ hybrids.**

Data are means ± SD (n=18 biologically independent samples). Statistical significance (**P* < 0.05; ***P* < 0.01) was determined by two-tailed Student’s *t*-test.

**References**

Chen J, Zeng B, Zhang M, Xie S, Wang G, Hauck A and Lai J (2014) Dynamic transcriptome landscape of maize embryo and endosperm development. *PLANT PHYSIOL* **166**:252-264.

Huang H, Xie S, Xiao Q, Wei B, Zheng L, Wang Y, Cao Y, Zhang X, Long T, Li Y, Hu Y, Yu G, Liu H, Liu Y, Huang Z, Zhang J and Huang Y (2016) Sucrose and ABA regulate starch biosynthesis in maize through a novel transcription factor, ZmEREB156. *SCI REP-UK* **6**:27590.

Walley JW, Shen Z, Sartor R, Wu KJ, Osborn J, Smith LG and Briggs SP (2013) Reconstruction of protein networks from an atlas of maize seed proteotypes. *Proceedings of the National Academy of Sciences* **110**:E4808-E4817.
